# Protein liposomes-mediated targeted acetylcholinesterase gene delivery for effective liver cancer therapy

**DOI:** 10.1186/s12951-021-00777-9

**Published:** 2021-01-22

**Authors:** Kai Wang, Fusheng Shang, Dagui Chen, Tieliu Cao, Xiaowei Wang, Jianpeng Jiao, Shengli He, Xiaofei Liang

**Affiliations:** 1 State Key Laboratory of Oncogenes and Related Genes, Shanghai Cancer Institute, Shanghai Jiao Tong University School of Medicine Affiliated Renji Hospital, Shanghai, 200032 People’s Republic of China; 2 Key Laboratory of Medical Molecular Virology (MOE/NHC/CAMS), School of Basic Medical Sciences, Shanghai Public Health Clinical Center, Shanghai Medical College, Fudan University, Shanghai, 200032 People’s Republic of China; 3 Institute of Translational Medicine, Shanghai University, Shanghai, 200444 People’s Republic of China; 4 Department of Hepatobiliary-pancreatic and Integrative Oncology, Minhang Branch, Fudan University Shanghai Cancer Center, Shanghai, 200240 People’s Republic of China; 5 Department of traditional Chinese medicine, Changzheng Hospital, Shanghai, 200001 People’s Republic of China

**Keywords:** Liposome, Transferrin, Acetylcholinesterase, Liver cancer, Gene therapy

## Abstract

**Background:**

Effective methods to deliver therapeutic genes to solid tumors and improve their bioavailability are the main challenges of current medical research on gene therapy. The development of efficient non-viral gene vector with tumor-targeting has very important application value in the field of cancer therapy. Proteolipid integrated with tumor-targeting potential of functional protein and excellent gene delivery performance has shown potential for targeted gene therapy.

**Results:**

Herein, we prepared transferrin-modified liposomes (Tf-PL) for the targeted delivery of acetylcholinesterase (AChE) therapeutic gene to liver cancer. We found that the derived Tf-PL/AChE liposomes exhibited much higher transfection efficiency than the commercial product Lipo 2000 and shown premium targeting efficacy to liver cancer SMMC-7721 cells *in vitro*. *In vivo*, the Tf-PL/AChE could effectively target liver cancer, and significantly inhibit the growth of liver cancer xenografts grafted in nude mice by subcutaneous administration.

**Conclusions:**

This study proposed a transferrin-modified proteolipid-mediated gene delivery strategy for targeted liver cancer treatment, which has a promising potential for precise personalized cancer therapy.

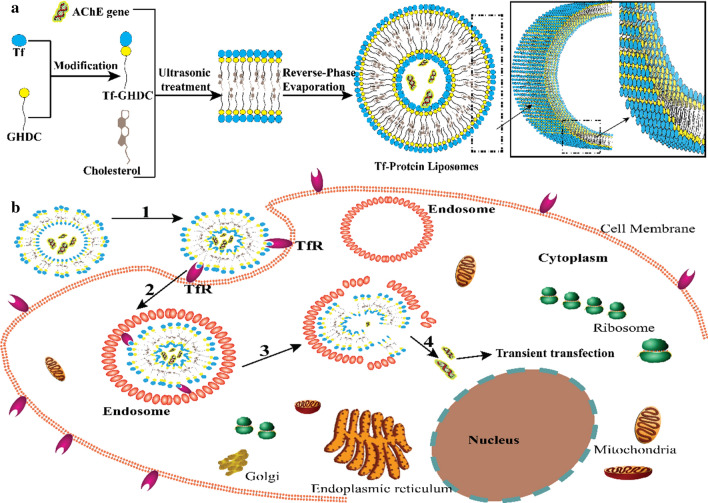

## Background

Liver cancer is a kind of common malignant tumor with poor prognosis [1–3]. Cancer is an extremely complex disease, usually caused by oncogene mutations [4–6]. Gene therapy provides a promising approach to the treatment of advanced cancer [7–10]. So far, more than 1500 cancer gene therapy programs have been confirmed for global clinical trials [11–13]. Therapeutic genes such as DNA and siRNA are easily degraded by enzymes in the blood, tissue or cytoplasm during transmission, and the biological half-life is very short [14, 15]. Moreover, therapeutic genes are highly hydrophilic macromolecular substances with strong negative charge under physiological conditions, so it is difficult to penetrate the cell membrane into the target cells [16–18]. Developing efficient gene delivery systems remains the biggest challenge for gene therapy [19, 20]. Due to its advantages of convenient preparation and mass production, non-viral vectors have attracted more and more attention from scholars all over the world [21]. Meanwhile, non-viral gene vectors have higher security for not cause obvious immune response and avoid the risk of neoplasm caused by viral vectors [22, 23]. Good biocompatibility, low cytotoxicity, simple preparation, low cost, good safety, strong tumor targeting, and high transfection efficiency are the core evaluation criteria for non-viral gene vectors [24–28].

So far four gene carrier drugs have been clinically licensed and these approved gene carrier drugs point the way for gene therapy and convey confidence [29]. As a type of drug delivery system, liposomes have great advantages in the delivery of anti-tumor drugs and genes. Onpattro was the first cationic liposomal nucleic acid drug approved for intravenous injection to treat nerve damage resulting from hereditary transthyretin-mediated amyloidosis by the European Union in 2018 [30]. Onpattro encapsulates siRNA in cationic lipid nanoparticles, and delivers drugs directly to the liver during infusion therapy to target and silence disease-causing transthyretin (TTR) [31]. It is known that transferrin (Tf) is a plasma protein responsible for transporting iron into cells via the transferrin receptor (TfR) [32–34]. TfR is overexpressed in many human tumors, and Tf-conjugated carrier can be used to selectively target drug delivery to cancer cells [35–38]. Many literature reports the content of the transferrin receptors in liver cancer tissues is higher than normal liver tissues [39, 40]. The tumor database information of Oncomine show that the expression of TfRs in liver cancer tissues was 2.780-fold higher compared to normal liver tissue (p < 0.01, AAdditional file 1: Figure S1). At the same time, the survival rate of the patients with high TfR expression was lower than the patients with low TfR expression (Additional file 1: Figure S2). Therefore, transferrin receptors can be a candidate active targeting site for liver cancer gene therapy.

**Fig. 1:**
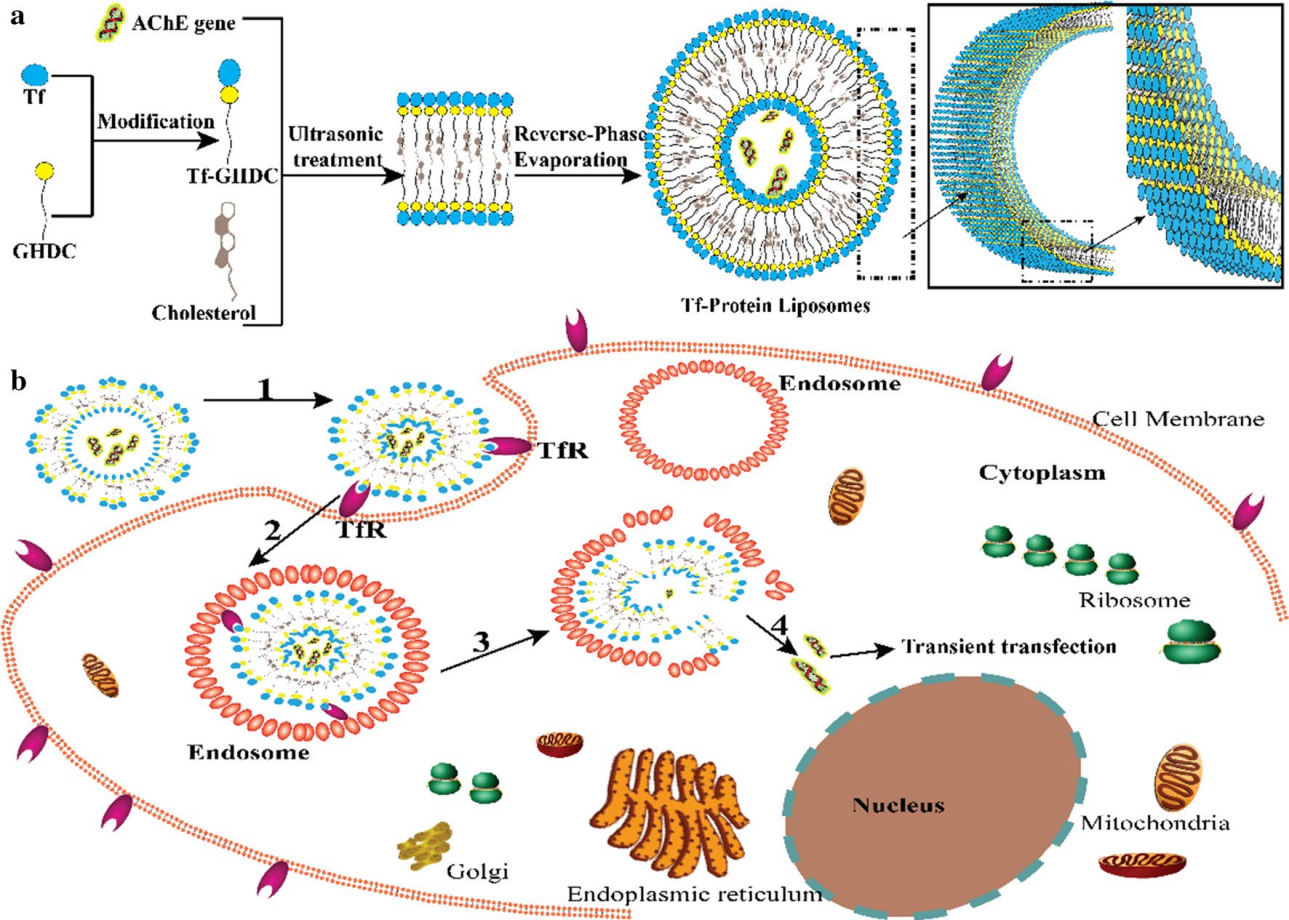
Diagrammatic drawing depicting the preparation and evaluation of Tf-PL/AChE. Gene-carrying transferrin liposome preparation procedure (**a**) and delivery strategy of transferrin liposome-carrying gene-targeted transferrin receptor-positive cells (**b**). The ‘proteoliposome’ interacts with the surface TfR of the targeted cell (1), and then enters the cytoplasm by endocytosis (2), released by lysosomal digestion (3), transient expression in the cytoplasm (4)

Acetylcholine (ACh) is involved in a variety of cell biological behaviors, such as cell proliferation, cell differentiation, cell migration and cellular immune response, so the accumulation of ACh in the tumor microenvironment directly or indirectly supports tumor growth [41]. Acetylcholinesterase (AChE) plays key role in the cholinergic system, and its dysregulation is involved in a variety of human diseases [42]. AChE can degrade the ACh and reduce the malignant development risk of liver cancer, thus AChE has been identified as a prognostic marker in liver cancer [41, 43, 44]. Oncomine database shows extremely high expression of ACh in various tumor tissues including liver cancer, p = 0.002 (Additional file 1: Figure S3), while AChE shows significant low expression in liver cancer p = 0.012 (Additional file 1: Figure S4). Therefore, AChE can be used as a gene therapy target for liver cancer. In this study, we for the first time to construct transferrin modified liposomes (Tf-PL) using transferrin-glycidyl hexadecyl dimethylammonium chloride (GHDC) instead of lipids, the transferrin was combined with GHDC to realize the hydrophilic and lipophilic modification, and then the transferrin liposome was prepared and used for gene loading. Tf-PL preparation was showed in Fig. 1a, transferrin was utilized to mediate the delivery of AChE gene to the cytoplasm via transferrin receptor-mediated endocytosis (Fig. 1b). In this study, the tumor targeting ability and growth inhibitory effect of Tf-PL/AChE were evaluated through a series of *in vitro* and *in vivo* experiments.

## Results and discussion

### Preparation and characterization of the protein liposomes

**Fig. 2:**
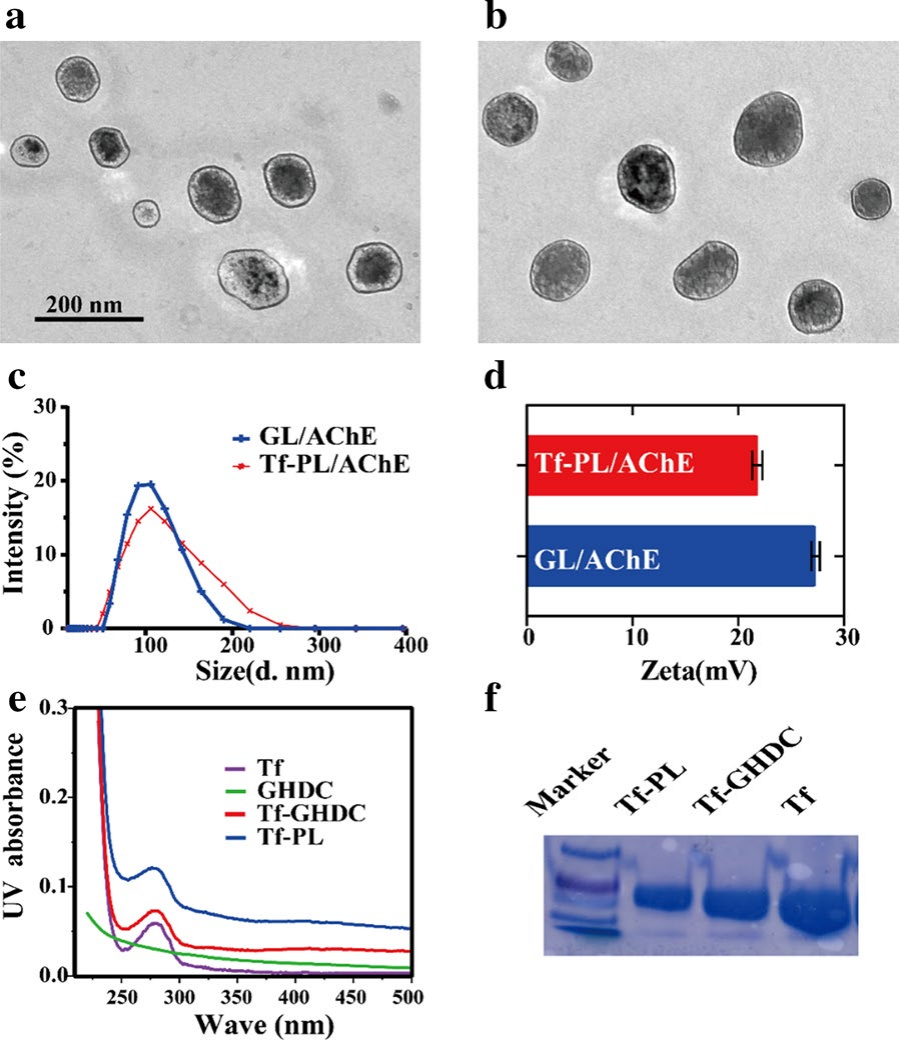
Performance characterization of transferrin liposomes. **a** TEM imaging of GL/AChE; **b **TEM imaging of Tf-PL/AChE (Scale bar = 100 nm); **c** dynamic light scattering analysis of particle size distribution; **d** dynamic light scattering analysis of the surface potential; **e** UV-vis absorption spectrum; **f** SDS-PAGE analysis of protein bands

In this study, the liposome was prepared by physically assembling method (Fig. 1a), the amphiphilic protein derivative modified by GHDC is different from the conventional protein-modified nanospheres or liposomes. In order to reflect the targeting capacity of Tf, non-Tf GHDC-liposomes (GL) were also prepared for comparison. The morphology of GL/AChE and Tf-PL/AChE was analyzed by transmission electron microscopy (TEM) (Fig. 2a, b). Dynamic light scattering (DLS) was used to evaluate the particle size and potential of GHDC-liposomes (GL) and Tf-PL. Figure 2c, d showed the particle size and potential data of liposomes in aqueous solution. The particle size of Tf-PL/AChE was 112.9 ± 4.5 nm (PDI = 0.149), the potential was 21.8 ± 0.5 mV, and the particle size of GL/AChE was 99.82 ± 2.2 nm (PDI = 0.118), and potential was 27.3 ± 0.4 mV (Table S5, Supporting Information). Both of the liposomes with double-layer skeleton structure with a regular spherical shape. The UV-vis spectrum in Fig. 2e showed that the intermediate Tf-GHDC and final Tf-PL have similar UV absorption curves with the Tf, indicating the successful Tf modification. The protein electrophoresis results indicated that Tf-PL has transferrin content (Fig. 2f).

The characteristic peaks of transferrin and GHDC were also observed in the nuclear magnetic H spectrum, and the long-chain methylene stretching vibration absorption peak was observed at 2922 cm^− 1^ and 2852 cm^− 1^ (Additional file 1: Figure S6). Suggesting the proteoliposome prepared in this study had a higher protein component. The optimized distribution ratio was determined by studying the effects of different distribution ratios on liposome size, charge, gene encapsulation efficiency (EL), and loading (DL) (Additional file 1: Table S1–4, Supporting Information). The loading of Tf-PL to AChE optimized to the distribution ratio was (6.31 ± 0.32)%, the encapsulation efficiency was (94.3 ± 1.01) %, the load corresponding to PL (6.07 ± 0.43)%, and the encapsulation ratio (91.2 ± 0.79) % (Additional file 1: Table S5). At the same time, the quantitative analysis of transferrin on the surface of Tf-PL performed by ELISA showed that the efficiency of transferrin modification on the surface of liposome was (88.7 ± 2.31) % (Additional file 1: Figure S7 and Table S5), suggesting most of the transferrin was distributed on the surface of proteoliposome.

### ***In vitro*gene release study and cytotoxicity of the proteoliposomes**

As a gene carrier, besides the high gene delivery efficiency, the Tf-PL also should have high stability during storage. The stability of GL/AChE and Tf-PL/AChE under different pH conditions was explored (Additional file 1: Figure S8). Additional file 1: Figure S8 A show the total release at different time points, and Additional file 1: Figure S8B show the gene release rate within 12 h. Compared with the rapid release of free AChE, GL/AChE and Tf-PL/AChE showed similar sustained release of AChE at pH 7.4 and pH 5.5; the burst release was not obvious, and the stability of Tf-PL/AChE complex was higher.

The Additional file 1: Figure S9 show the *in vitro* cytotoxicity of the prepared proteoliposomes. The results indicated that the relative survival rate of normal liver HepZJ cells and SMMC-7721 cells were still high when the concentration of GL and Tf-PL reached 500 µg/mL after 48 h of culture. Herein, the proteolipid prepared in this study has good biocompatibility and low cytotoxicity, which lays a foundation for subsequent applications.

### ***In vitro*cellular uptake and transfection efficiency study**

**Fig. 3:**
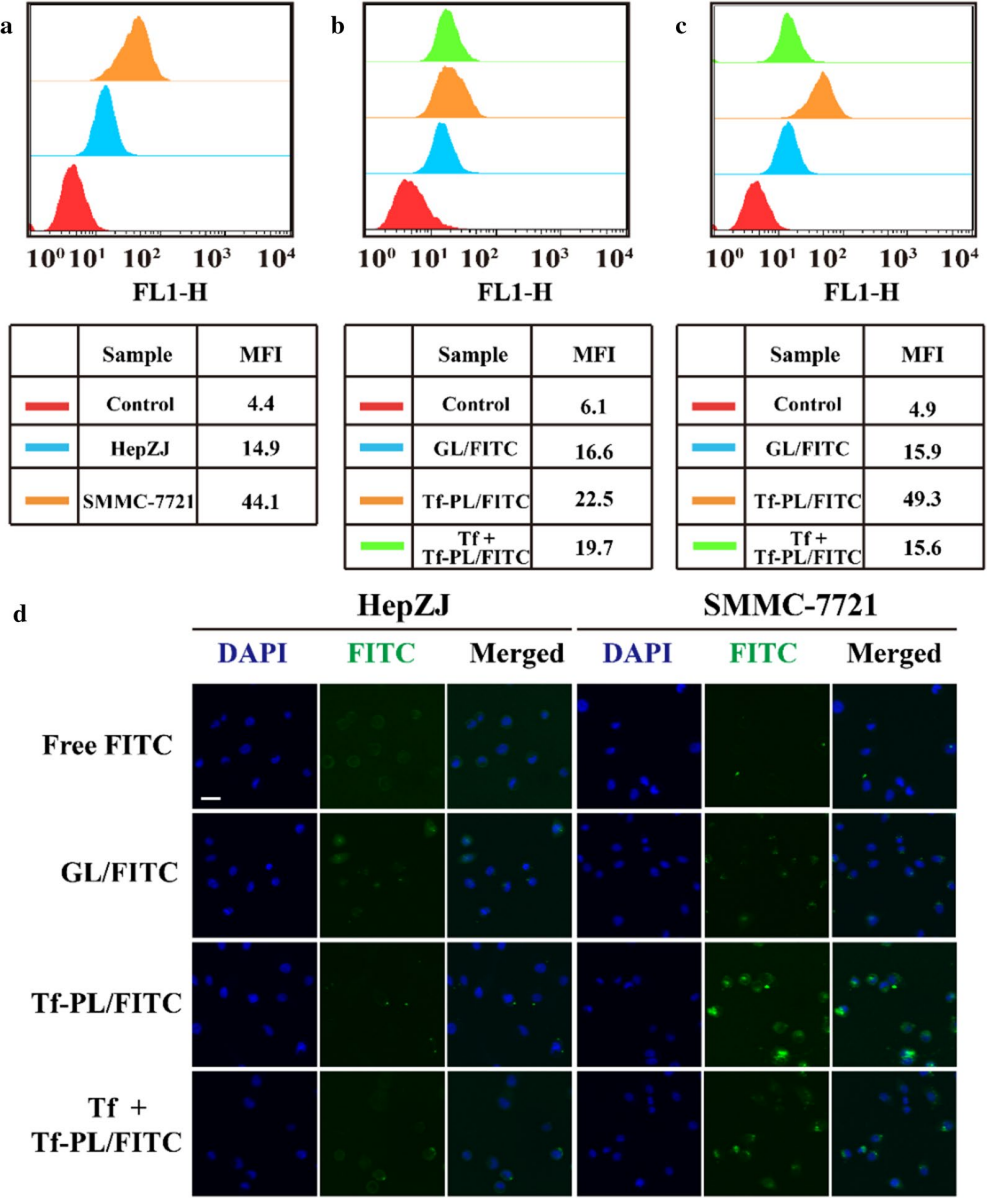
Transferrin receptor as a drug delivery target for liver cancer cells. **a** Flow cytometry analysis of TfR expression in SMMC-7721 and HepZJ cells; **b** Flow cytometry evaluation HepZJ cell uptake of GL and Tf-PL. **c** Flow cytometry evaluation SMMC-7721 cell uptake of GL and Tf-PL. **d** Confocal microscope evaluation cellular uptake study of GL and Tf-PL. Scale bar = 10 µm

To determine whether the TfR could be a therapeutic target for the treatment of liver cancer, we compared the expression of TfR between liver cancer (N2 = 225) and normal liver tissue (N1 = 220) by using the online database Oncomine. We found that the expression of TfR was significantly increased in liver cancer specimens, which was 2.780 times the normal specimens (Additional file 1: Figure S1). In order to further investigate the expression of TfR in cells, we selected liver cancer cells and immortalized liver cell lines currently preserved in the laboratory, and detected TfR expression by Western blot. The results in Additional file 1: Figure S5 show that compared with normal liver cells, liver cancer cell lines express higher levels of TfR. TfR may be a potential drug delivery target for the treatment of liver cancer. Then, we compared the expression of TfR in HepZJ and SMMC-7721 cells by flow cytometry. As shown in Fig. 3a, the flow cytometry results showed that the TfR expression of SMMC-7721 was as much as 7.36 times of HepZJ cells, the expression difference between the two cells was significant (p < 0.01), which can be used for further experimentations. To study the interaction between liposomes and cell surface receptors, and assess the ability of Tf-PL to specifically bind to TfR and trigger receptor-mediated internalization of liposome delivery in TfR-positive cells, we compared binding efficiency of FITC-labeled GL and Tf-PL in SMMC-7721 cells. As shown in Fig. 3c, SMMC-7721 cells showed significant higher endocytosis of Tf-PL/FITC than GL/FITC, but there was no significant difference in HepZJ cells (Fig. 3b). In order to study whether Tf-PL/FITC was taken up through TfR-mediated endocytosis, the SMMC-7721 cells were treated with Tf for 6 h before the Tf-PL/FITC added. Confocal microscopy shows that the FITC fluorescence was significantly reduced, so the endocytosis of Tf-PL/FITC by SMMC-7721 cells was disturbed by the competitive combination of Tf. The confocal microscopy result (Fig. 3d) indicated that Tf-PL was taken up through TfR-mediated endocytosis. We also analyzed the subcellular localization of Tf-PL after endocytosis by cells, the results of confocal experiment showed that the endocytic Tf-PL were mostly distributed in cytoplasm and partly in lysosomes (Additional file 1: Figure S10), so Tf-PL would not permanently trap in lysosomes.

**Fig. 4:**
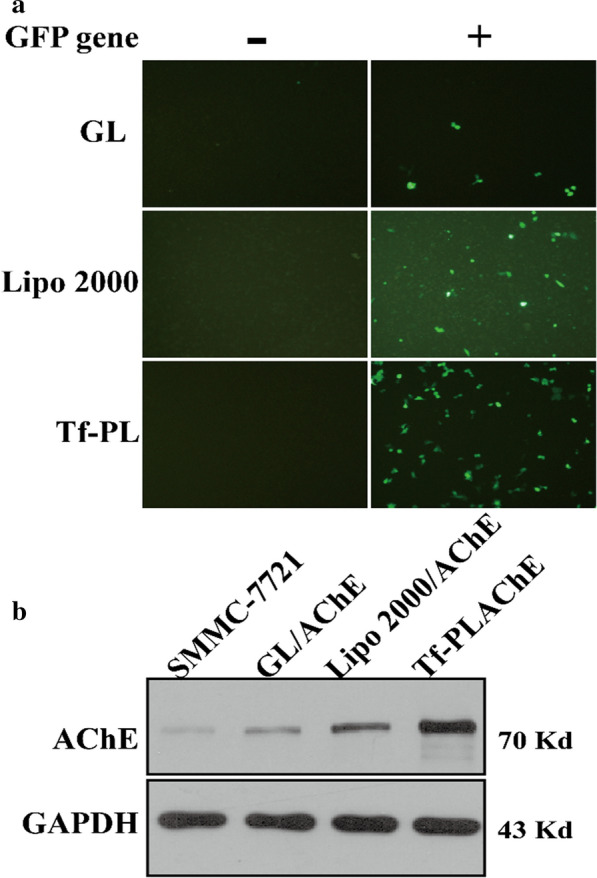
Analysis of gene transfection efficiency. **a** Fluorescence intensity of EGFP expression; **b** Western Blot analysis of AChE expression in different gene carrier treatment

To evaluate the efficiency of Tf-PL as a gene vector for gene delivery to cells, we compared the transfection efficiency of Tf-PL with commercial transfection reagent Lipo 2000 on SMMC-7721 cells. By comparing the amount of GFP fluorescence at 36 h and 48 h, we found that the gene transfection efficiency of Tf-PL is significantly stronger than Lipo 2000 (Fig. 4a). The AChE gene delivery efficiency of Tf-PL was analyzed by Western blot experiment, the Western blot results showed SMMC-7721 cells treated with GL/AChE, Lipo 2000/AChE, Tf-PL/AChE showed an increase in the expression of AChE, and SMMC-7721 cells treated with Tf-PL/AChE showed the most significant increase in AChE expression. Based on the above research results, it is concluded that the Tf-PL is suitable to be used as a vector for SMMC-7721 cells gene therapy.

### ***In vitro*tumor cell proliferation inhibition study**

**Fig. 5:**
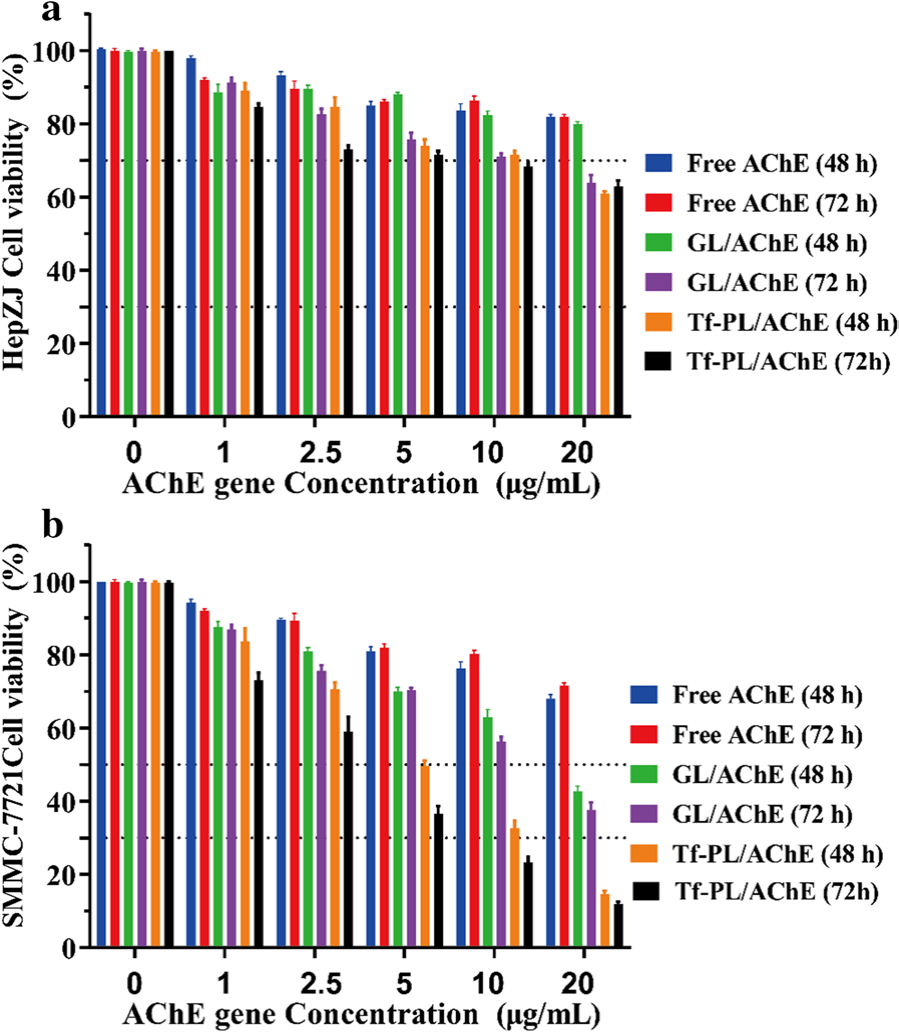
*In vitro* cytotoxicity and cell migration of AChE treatment study. **a** AChE treatment study on HepZJ cells; **b** AChE treatment study on SMMC-7721 Cells

Additional file 1: Figure S3, 4 shows that an overexpression of ACh and a low expression of AChE were detected in liver cancer cells. Therefore, we measured the effect of ACh and AChE on the proliferation of SMMC-7721 cells by CCK-8 assay. As shown in Additional file 1: Figure S11 A, ACh could significantly promote the proliferation of SMMC-7721, with the increased concentration and prolonged action time of ACh, the proliferation promotion effect is more obvious. On the contrary AChE could significantly inhibit the proliferation of SMMC-7721, and the inhibition of proliferation was more obvious with the increase concentration and prolonged action time of AChE (Additional file 1: Figure S11 B). Therefore, this study demonstrated that the overexpression of AChE is a theoretically strategy to inhibit the proliferation of liver cancer cells. Then the effect of AChE gene therapy on liver cancer SMMC-7721 cells was studied. It was found that GL/AChE and Tf-PL/AChE inhibited the growth of SMMC-7721 cells in a concentration- and time-dependent manner. Free AChE gene had little effect on the proliferation of SMMC-7721 cells and HepZJ cells (Fig. 5). Tf-PL/AChE showed the highest cytotoxicity with IC50 values of 4.25 µg/mL (48 h) and 3.45 µg/mL (72 h), respectively (Fig. 5b). Thus, Tf modification could significantly enhance the uptake of Tf-PL/AChE by liver cancer SMMC-7721 cells, Tf-PL delivered more AChE to cells and subsequently inhibited cell growth.

### ***In vitro*cell migration and wound healing study**

Cell migration plays an important role in tumor growth and metastasis, so the role of Tf-PL/AChE on the migration of SMMC-7721 cells was assessed by transwell migration and scratch experiments (Additional file 1: Figure S12, 13). Although obvious migration of the SMMC-7721 cells without any treatment was observed. The migration of SMMC-7721 cells treated with Tf-PL/AChE and GL/AChE significantly reduced. and Tf-PL/AChE was the most effective, the inhibition rate was 45.1 %, p < 0.05. These observations indicate that Tf-PL/AChE treatment can effectively block the migration of SMMC-7721 cells. In the wound healing experiment, 48 h after cell scratch, the control group, free AChE and GL/AChE group showed significant cell healing, while Tf-PL/AChE Group SMMC-7721 cell wound healing was significantly delayed. The above results indicate that Tf-PL/AChE treatment significantly affected the migration and proliferation of SMMC-7721, Tf-PL could improve the effect of gene therapy.

### Cell cycle and apoptosis study

**Fig. 6:**
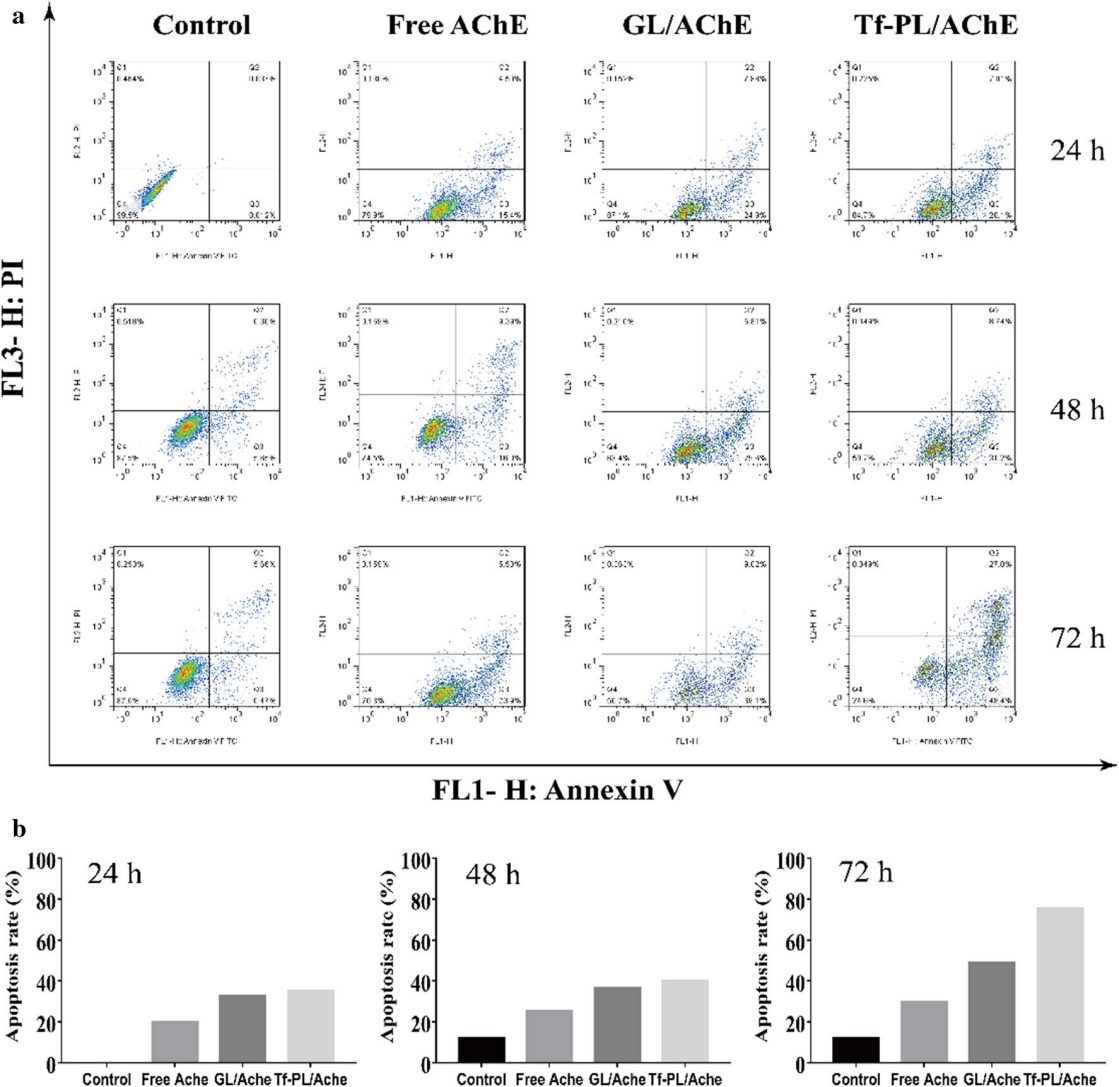
Cell apoptosis analysis. **a** Flow cytometric analysis and **b** quantification of cell apoptosis analysis in SMMC-7721 cells following treatment with Control, Free AChE, GL/AChE, Tf-PL/AChE for 24, 48 and 72 h at an equivalent AChE concentration of 2.5 µg/mL

To further investigate the anti-proliferative effect of Tf-PL/AChE in hepatocarcinoma cells, SMMC-7721 cells were treated with Tf-PL/AChE for 24, 48, and 72 h, respectively. Then the cell apoptosis and cell cycle of SMMC-7721 cells were detected by flow cytometry. The results showed that the GL/AChE and Tf-PL/AChE could induce apoptosis in SMMC-7721 cells in a time-dependent manner (Fig. 6a). Compared with GL/AChE treated cells, the apoptotic rate of Tf-PL/AChE treated cells was significantly increased at 48 and 72 h (Fig. 6b).

**Fig. 7:**
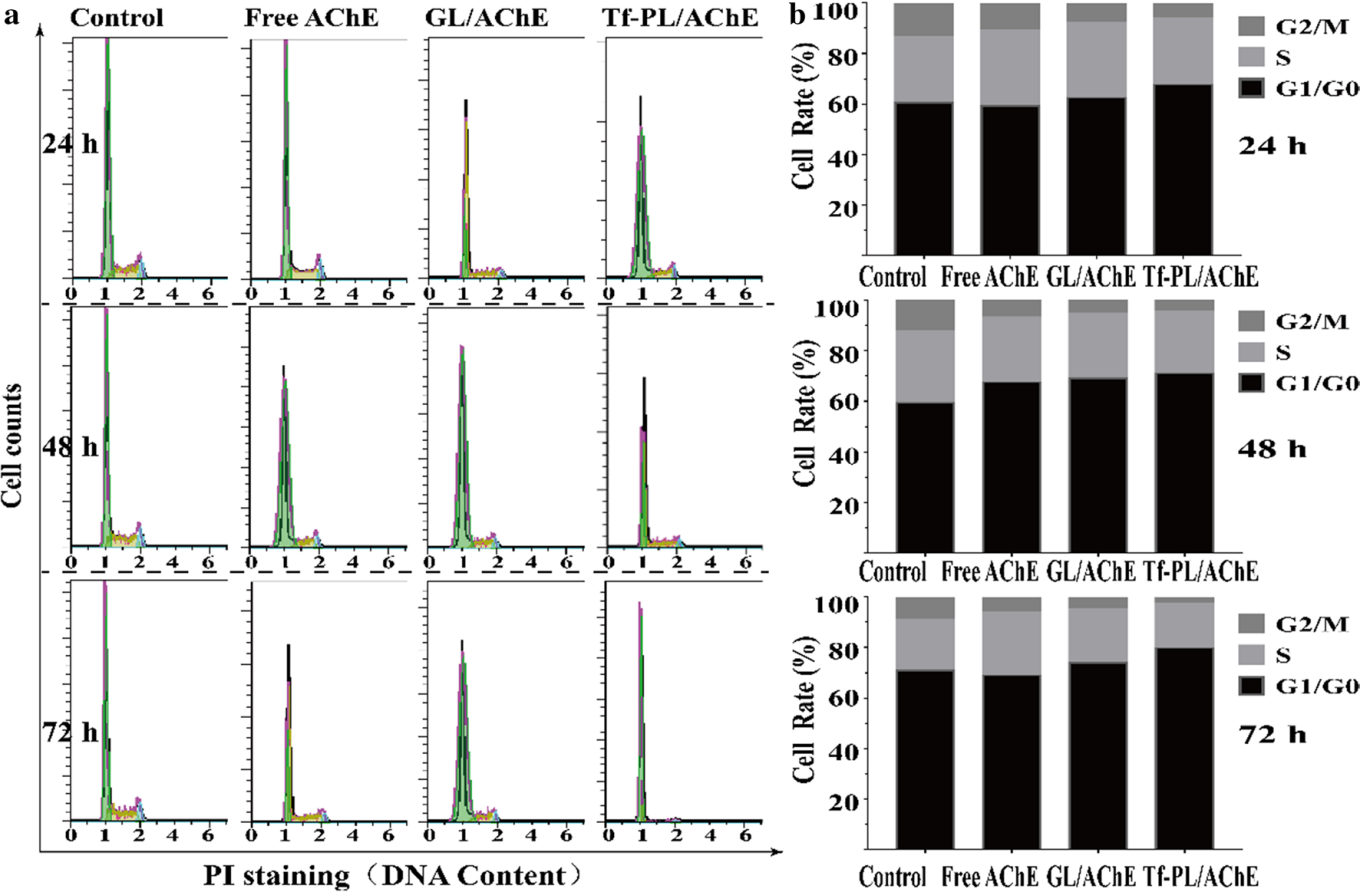
Cell cycle analysis. **a** Flow cytometric analysis and **b** quantification of cell cycle distribution in SMMC-7721cells following treatment with Control, Free AChE, GL/AChE, Tf-PL/AChE for 24, 48, and 72 h at an equivalent AChE concentration of 2.5 µg/mL

At the same time, the cell cycle analysis was also performed. As shown in Fig. 7, SMMC-7721 cells showed significant G0/G1-S phase accumulation after AChE gene therapy, while the G2/M phase decreased. This indicated that the transition from the S phase to the G2/M phase can be disrupted in cells treated with the transferrin liposome carrying the AChE gene, with the Tf-PL/AChE group having the best effect. Therefore, Tf-PL/AChE-treated cells showed an inhibited growth of liver cancer cells by inducing apoptosis and cell cycle arrest.

### ***In vivo*imaging distribution study**

**Fig. 8:**
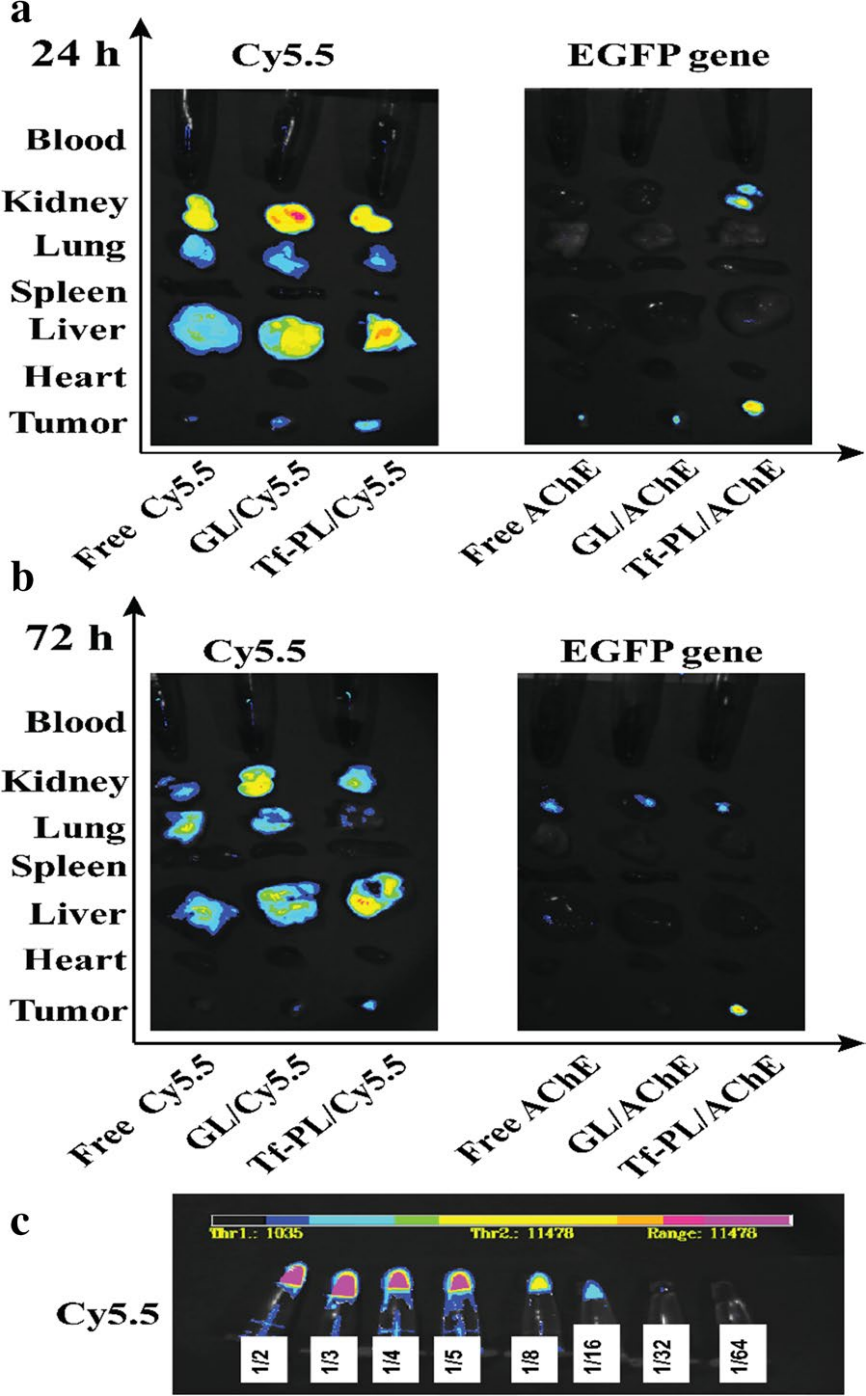
*In vivo* distribution of Tf-PL. **a** fluorescence distribution at 24 h; **b** fluorescence distribution at 72 h; **c** fluorescence intensity reference

Effective targeting of tumor cells is essential for inhibiting tumor growth. To investigate the tumor-targeting efficiency of Tf-PL/AChE *in vivo*, we established a nude mouse model of SMMC-7721 tumors. Real-time fluorescence imaging of nude mouse organs and tumors was performed by injecting a near-infrared dye (Cy5.5)-labeled liposomes into the tumor-bearing nude mice, and the target effect of the Tf-PL/AChE was studied by the distribution of fluorescent signals (Fig. 8). The fluorescence image of Cy5.5 showed that after 24 h of injection, the fluorescence signal was mainly found in the kidney, lung, liver, tumor, and peripheral blood. Moreover, the fluorescence signal of mouse tumors in the Tf-PL group was stronger than the other groups. After 72 hours, the fluorescence signals in the kidney, lung, liver, tumor, and peripheral blood were significantly decreased. There was still obvious Cy5.5 signal distribution in the tumor of the Tf-PL group, but the intratumoral signal of the PL group was significantly decreased. At the same time, green fluorescent protein (GFP) expression was studied to indirectly mimic the expression of the acetylcholine ester gene to analyze the targeted delivery of the Tf-PL-carrying gene to the tumor. There was a significant GFP fluorescence signal at the tumor site in mice, 24 h or 72 h. Based on the above experimental results, it can be proved that the Tf-PL group has extremely high intensity and distribution in tumors, so Tf-PL can effectively target liver cancer.

### ***In vivo*antitumor efficacy study**

**Fig. 9:**
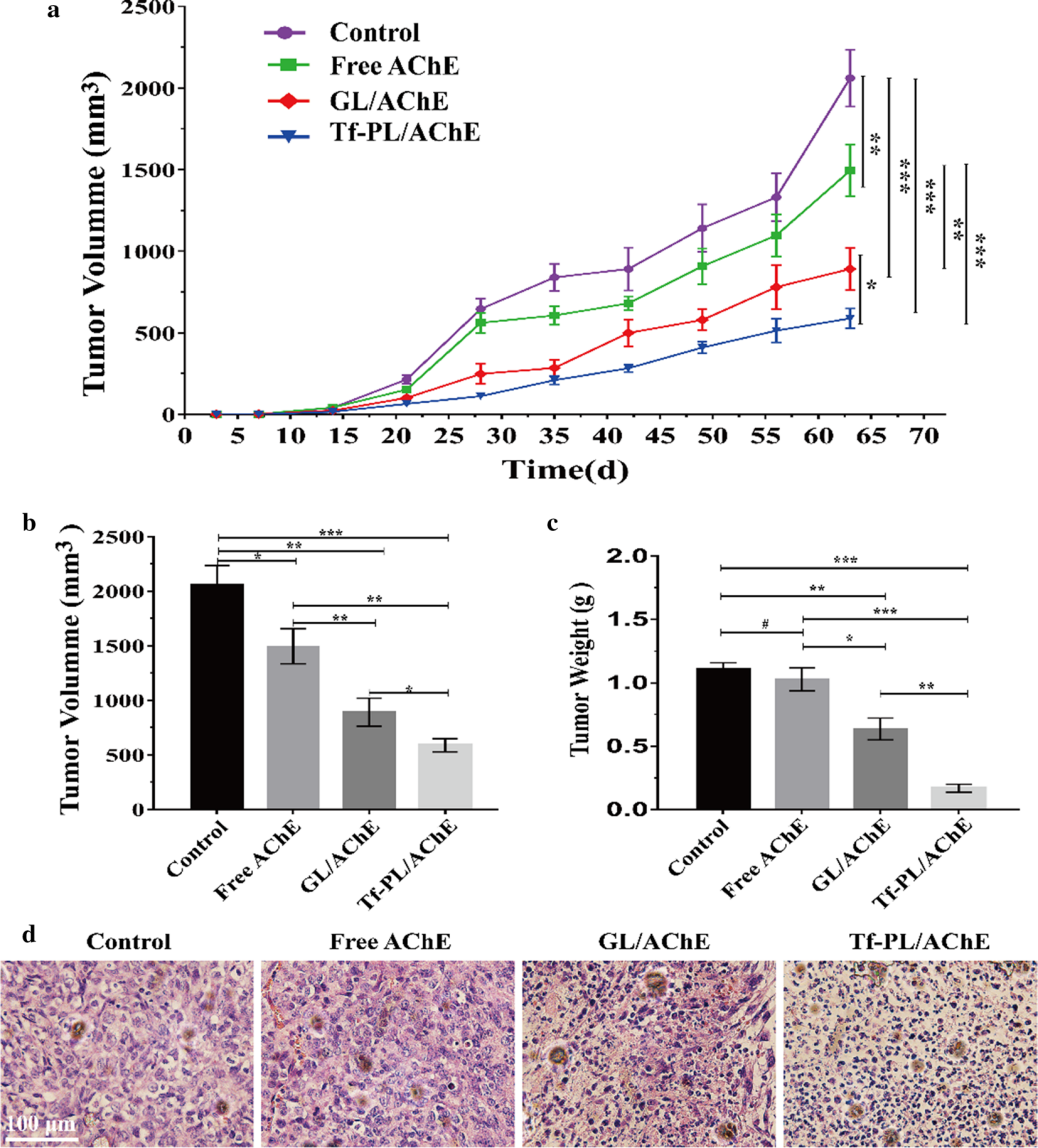
Analysis of tumor treatment effect. **a** The relative tumor size of the subcutaneous model changed with time; **b** Image of tumor size in each group; **c** Relative tumor volumes as a function of time; **d** HE staining of tumor tissue from different tumor treatment groups

In this study, a nude mouse xenograft model of SMMC-7721 tumor was established, and different therapy treatments were given to analyze the anti-tumor effects of different groups. Mice bearing SMMC-7721 tumors were injected with normal saline, free AChE, GL/AChE, and Tf-PL/AChE every 7 days for 4 consecutive injections. As shown in Fig. 9a, free AChE had slight efficiency in inhibiting tumor growth, tumor growth in mice treated with GL/AChE and Tf-PL/AChE was significantly inhibited. Especially, the mice treated with Tf-PL/AChE had the most pronounced inhibitory effect on tumor growth compared to the other groups (Fig. 9a and Additional file 1: Figure S14). The final weight and volume of the tumor in the Tf-PL/AChE group were (0.25 ± 0.12) g and (517.14 ± 112.63) mm^3^, respectively (Fig. 9b, c). The tumor suppression effect of free AChE was 7.81 %, tumor growth inhibition was inefficient. However, the tumor weight inhibition rate of Tf-PL/AChE was 77.47 %, while that of GL/AChE group was only 48.21 %, Tf-PL/AChE effectively inhibited the growth of liver cancer cells and reduced the weight of the tumor. These results supported the superior antitumor efficacy of Tf-PL/AChE treatment *in vivo*. As shown in Fig. 9d, the HE staining results showed the tumors in the GL/AChE and Tf-PL/AChE groups showed mild staining and a large area of blank, in Tf-PL/AChE group tumor tissue showed a large area of necrosis, so the tumor tissue necrosis was very serious. The tumor tissues of control and the Free AChE groups were darker, showed identifiable tissues. Taken together, the Tf-PL/AChE could effectively inhibit tumor growth.

Currently, non-viral vectors have been widely used for gene therapy [43, 44]. In order to achieve targeted delivery of therapeutic drugs to tumor cells, receptor-mediated active targeting has been generally adopted in recent years, such as the presence of asialoglycoprotein receptors on liver parenchymal cell membranes; mannose receptors are distributed on non-parenchymal cell membranes. According to many studies both at home and abroad, it has been suggested that the content of transferrin receptor in liver cancer tissue is significantly higher than that in adjacent normal tissue [45, 46], as shown in Additional file 1: Figure S1. Therefore, Tf-modified liposomes can increase the therapeutic gene uptake by liver cancer cells with high TfR expression on the cell membrane surface, thus the therapeutic genes can specifically act on liver cancer cells, reducing the possibility of toxic side effects. Our study describes a method for easily preparing a neutral targeting gene delivery system by complexing cationic nanoparticles with a therapeutic plasmid by electrostatic interaction. The obtained neutral targeting gene delivery system provides a robust and flexible non-viral platform for mediating cancer gene therapy for intravenous administration.

In this work, we attempted to directly construct protein liposomes using protein derivatives by amphiphilic modification of the tumor-targeting protein-transferrin, and by *in vitro* and *in vivo* delivery of the gene. The effect evaluation confirmed the significance of the effectiveness and targeted effect of the constructed gene/proteoliposome. In order to study the performance of Tf-modified AChE gene-carrying proteoliposomes, we conducted a series of cell experiments in vitro. Qualitative and quantitative analysis showed that Tf-PL enters SMMC-7721 cells more significantly than non-targeted liposome GL, significantly increasing the fluorescence intensity in targeted liver cells. The TfR competition experiment showed that the uptake of Tf-PL by SMMC-7721 cells after the TfR receptor was occupied by Tf was significantly reduced, so Tf can competitively inhibit the targeted endocytosis mediated by the TfR receptor and Tf-PL mainly depends on the TfR mediated endocytosis into liver cancer cells. These results indicate that Tf-modified AChE-loaded proteoliposomes have good active targeting properties.

Then analyze the tumor cell inhibitory ability of Tf-guided targeting liposomes. Cytotoxicity test results show that compared with Free AChE and GL/AChE groups, Tf-PL/AChE has a more obvious inhibitory effect on the proliferation of liver cancer cells, and is concentration- and time-dependent, which may be significantly related to Tf-PL/AChE. Apoptosis and cell cycle arrest in G0/G1-S phase is related (see Figs. 6 , 7). Cell migration experiments and scratch repair experiments show that Tf-PL/AChE has a better therapeutic effect than Free AChE and GL/AChE treatments, and can significantly inhibit cell proliferation and cell migration. This may be due to the modification of Tf on the surface of liposomes, receptor-mediated endocytosis makes it easier for liposomes to enter cells, and the accumulation of therapeutic genes in cells is higher, thereby enhancing the inhibitory effect on tumor cells.

*In vivo* imaging of small animals showed that Tf-PL/Cy5.5 liposomes still had a strong fluorescence intensity at the tumor site 72 hours after receiving fluorescence injection; while Cy5.5 basically did not see fluorescence at the tumor site. In the in vivo anti-tumor experimental study, we found that compared with Free AChE and GL/AChE, Tf-PL/AChE can effectively inhibit the growth of subcutaneous transplanted tumors in mice bearing SMMC-7721 liver cancer. At the end of the experiment, the tumor weight inhibition rate of Tf-PL/AChE was 77.47 %, while that of GL/AChE group was only 48.21 %. Our data suggest that the AChE gene is an effective therapy gene for the treatment of liver cancer and Tf-PL shows promising clinical applications value.

## Conclusions

In this work, we put forward a strategy for the delivery of the AChE gene by the transferrin modified liposome targeting TfR on the surface of liver cancer cells for liver cancer therapy. This strategy provides an alternative approach to replace the conventional virus mediated gene carrier for cancer therapy, which can effectively bypass the biosafety problems that live viruses may cause. The present gene delivery system has good blood compatibility and degradability, low toxicity, good tumor targeting ability, and high transfection efficiency. Furthermore, ectopic expression of the AChE gene can significantly inhibit the proliferation of liver cancer cells in xenograft model. The proteolipid prepared directly from Tf enhances the therapeutic gene transfected into the hepatoma cells, suggesting a potential liver cancer delivery system. In conclusion, the strategy of combining the transferrin liposome and AChE gene provides a new idea for gene therapy of liver cancer.

## Methods

### Materials

#### Reagents and kits

N, N-Dimethyltetradecylamine (≥ 99 %) was purchased from Feixiang Corporation. Fluorescein isothiocyanate I (FITC); cholesterol and other molecular biology reagents were purchased from Sigma, USA. The AChE gene was deposited by the National Laboratory of Oncogenes and Related Genes of the Shanghai Cancer Institute (sequence number: NG007474.1). Annexin V-FITC/PI apoptosis kit (70-AP101-100) for FACS and BCA protein quantitative Kit (70-PQ0011) were purchased from MultiSciences (China). Lipofectamine 2000 (Lipo 2000) was purchased from Invitrogen (USA). 1,2-dioleoyl-sn-glyceryl-3-phosphoethanolamine (DOPE), 1,2-distearoyl-sn-glyceryl-3-phosphoethanolamine-N- [(polyethylene glycol)- 2000] (DSPE-PEG) was purchased from Avanti (USA). Fetal bovine serum, penicillin/streptomycin, Dulbecco’s modified Eagle’s medium, DMEM were purchased from Gibco, USA. RIPA lysis buffer, DMSO, and PMSF were purchased from Shanghai Sangon Biotech Co., Ltd. The Transwell chamber was purchased from Corning (USA). Mouse anti-human AChE monoclonal antibody, mouse anti-human TfR monoclonal antibody was purchased from Santa Cruz, USA. Goat anti-mouse IgG (H + L)-HRP, goat anti-rabbit IgG (H + L)-HRP was purchased from Abcam (USA). Epichlorohydrin (≥ 99 %), absolute ethanol, isopropanol (≥ 99 %), chloroform (≥ 99 %), ethylenediaminetetraacetic acid (EDTA, 99.9 %), epichlorohydrin, and other biochemical reagents were analytical grades and are purchased from Sinopharm. Lyso-Tracker Red and DAPI were purchased from Shanghai Beyotime Biotechnology. Glycidyl hexadecyl dimethylammonium chloride (GHDC) and other polymers are synthesized and stored by our laboratory.

#### Preparation of AChE plasmid

The therapeutic acetylcholinesterase (AChE) plasmid was obtained from the National Laboratory for Oncogenes and Related Genes, Cancer Institute of Shanghai JiaoTong University (sequence:NG007474.1). Plasmid amplification was achieved by transforming competent E. coli and enlarging the number of E. coli in large quantities, and the plasmid was extracted using the Qiagen EndoFree Plasmid Mega Kit (Qiagen, Hilden, Germany). After passing the test, the plasmid was then dissolved in sterile endotoxin-free water and stored at − 20 °C for later use.

#### Cell culture and transfection experiments

Immortalized human liver normal cells HepZJ cells, liver cancer cell line SMMC-7721 cells were preserved by the laboratory. And cultured in Dulbecco’s Modified Eagle Medium (DMEM) supplemented with 10 % fetal bovine serum (FBS), 100 U/mL^-1^ penicillin, and 100 µg/mL streptomycin in a humidified incubator with 5 % CO_2_ at 37 °C. SMMC-7721 cells were seeded at 2 × 105 cells/well in a 6-well plate (Corning Inc., NY, NJ, USA) in 2 mL of complete medium. After 24 hours of incubation, the medium in each well was replaced with 2 mL of fresh serum-free medium. The pVAX-GFP (pGFP) reporter plasmid was maintained at 2 µg per well, while the mass ratios of GL/pGFP, Tf-PL/pGFP, and Lipo 2000/pGFP were 25:1. The serum-free medium was then replaced with a complete medium after 6 h. Then, the cells were cultured for an additional 48 hours at 37 °C. The expression of GFP was visualized by an Olympus IX 71 inverted fluorescence microscope (Olympus Corp., Tokyo, Japan). Cell suspensions were harvested and analyzed by flow cytometry (BD Biosciences, San Jose, CA, USA) to determine transfection efficiency.

#### Liver cancer cell lines and animal experiments

SMMC-7721 cells in Dulbecco’s Moded Eagle’s medium containing 10 % fetal bovine serum (FBS) (GIBCO BRL, Grand Island, NY, USA). The concentration of penicillin and streptomycin in the medium was 50 µg/mL, respectively; and the cells were cultured at 37 °C in a humidified environment containing 5 % CO_2_. Female BALB/c-nu nude mice (6–8 weeks old; body weight, 18–20 g) were obtained from Shanghai Experimental Animals Inc. (SLAC; Shanghai, China) and maintained under conditions free of specific pathogens. All animal experiments were conducted in accordance with the guidelines set by the Animal Care and Use Committee of Shanghai Jiao Tong University School of Medicine (Shanghai, China).

#### Synthesis of Tf-GHDC

Tf-GHDC is synthesized by conjugating Tf with GHDC. Accurately weigh 20 mg of Tf dissolved in 20 mL of double deionized water, add 20 mg of GHDC, and stir gently to form a conjugate. The resulting solution was incubated at 37 °C for 24 h to allow the reaction to proceed. Unreacted GHDC was separated from the conjugate by dialysis against ddH_2_O for 36 hours. Nuclear magnetic resonance analyzer was performed to obtain spectral absorption peaks of Tf, CHDC, and Tf-GHDC to compare changes in Tf before and after conjugation.

#### Synthesize the Tf-PL/AChE nanoparticles

The main steps of preparing Tf-PL by thin film dispersion method are as follows:

1. Accurately weigh the lipid material with a molar ratio of DOPE:CHOL: DSPE-PEG = 3:1:0.4 and dissolve it in an appropriate amount of chloroform solution;
2. Place the solution in a spherical bottle at room temperature for 5–10 min, and then remove the chloroform by rotary evaporation under reduced pressure to form a colorless transparent film;
3. Vacuum the spherical flask overnight to completely remove organic solvents;
4. Take a certain amount of Tf-GHDC lyophilized powder dissolved in PBS buffer and add it to the lipid film in (3), and hydrate it in a 35 °C water bath for 4 hours;
5. Ultrasound the above suspension in a water bath with an ultrasonic power of 100 W, with 5 S interruptions every 10 S, and repeat 100 times to obtain Tf-PL/AChE.
6. The liposome precipitate was obtained by centrifugation, resuspended in ultrapure water, and stored at 4 °C.

The preparation process of transferrin liposomes without plasmids or drugs is the same as that of Tf-PL/AChE, except that only Tf-GHDC lyophilized powder PBS solution is added for hydration. The preparation of other liposomes is the same as that of Tf-PL/AchE-Dox, except that GHDC lyophilized powder is replaced by Tf-GHDC lyophilized powder. The preparation process of fluorescently labeled transferrin liposomes is the same as that of Tf-PL/AchE-Dox, except that FITC-Tf-GHDC lyophilized powder is replaced by Tf-GHDC lyophilized powder.

#### Characterization of Tf-PL/AChE

The average particle size, size distribution, and zeta potential of the proteoliposome were determined using a Malvern Zetasizer (Nano-ZS 90, Malvern Instruments Limited, UK) based on quasi-elastic light scattering at 25 °C. The morphology and shape of the liposomes were imaged by TEM. Before imaging, the liposomes were coated on a carbon-coated copper grid, stained with 4 % uranyl acetate for 10 min, and allowed to dry. TEM was carried out using a 7650 TEM (Hitachi; Kyoto, Japan) at 120 kV. Agarose gel electrophoresis experiments were performed with different weight ratios of Tf-PL and DNA. The prepared transferrin liposome solution was placed in an ultrafiltration centrifuge tube, centrifuged at room temperature for 15 min at 8 000 r/min, and the flow-through was removed and then using an anti-human transferrin ELISA kit to qualify the dose according to the manufacturer’s instructions. The conjugation efficiency of Tf was calculated. The calculation formula is: CE% = (transferrin addition amount - transferrin outflow amount) / transferrin addition amount × 100 %. In the same way, non-targeted liposomes (GL) were prepared only with GHDC and cholesterol (Chol). FITC and Cy5.5 labeled liposomes were prepared by adding the desired amount of FITC to the lipid organic solution before the solvent evaporation step and adding Cy5.5 to ddH_2_O before the hydration step.

#### Characterization of the transgenic performance of proteoliposome

The appropriate amount of proteoliposome was taken and demulsified with methanol. Nanodrop 2000 was used as the main absorption peak of nucleic acid with 260 nm ultraviolet absorption peak. The gene load (DL) of the liposome and the encapsulation efficiency (EE) of the liposome to the gene were determined according to the formula. The calculation formula is: DL% = (total amount of gene - unencapsulated free gene) / total amount of system × 100 %; EE% = (total amount of gene - unencapsulated free gene) / gene × 100 %. The stability analysis of the *in vitro* gene of the proteoliposome carrying the gene was determined using a dialysis method. Briefly, 2 mL of plasmid-loaded liposomes were suspended in a dialysis bag with a molecular weight cut-off of 12 kDa and dialyzed against 18 mL PBS (pH 7.4) containing 0.1 % Tween-80 for 7 days (v/v) On a horizontal shaker (100 rpm) at 37 °C. 2 mL aliquot was taken at predetermined intervals and replaced with an equal volume of fresh medium. The DNA content of the samples collected at each time point was measured using Nanodrop 2000.

#### Expression of transferrin receptor and acetylcholinesterase in hepatocellular carcinoma cell lines

The normal liver cells and three human hepatoma cell lines were selected, and the expression level of TfR on the cell membrane was analyzed by Western Blot. The expression of acetylcholinesterase in normal liver cells and three human hepatoma cell lines were analyzed. The cells were collected in 1.5 ml tubes, washed twice with PBS, then 0.1 ml RIPA lysis buffer containing 1 mM PMSF was added, and then placed on ice for 30 minutes. The supernatant was obtained by centrifugation at 13,000 rpm for 15 minutes at 4 °C. Subsequently, the protein concentration was determined by BCA protein quantification. A total of 20 µg of protein sample was separated on a 12 % SDS-PAGE gel and then transferred to a PVDF membrane which was blocked in 5 % skim milk for 1 hour. Membranes were incubated with mouse anti-human AChE monoclonal antibody or mouse anti-human TfR monoclonal antibody (1:500) overnight at 4 °C and washed three times with PBST followed by goat anti-mouse IgG (H + L)- HRP was incubated for 2 hours at room temperature. Finally, ECL luminescence is used for detection.

#### Cellular uptake and localization of liposomes in SMMC-7721 cells

SMMC-7721 cells were incubated with proteoliposomes at a series of FITC concentrations (0.33, 1, and 2 nM) for 2 hours at 37 °C and then washed three times with PBS. Cellular uptake of FITC-labeled proteoliposomes was qualitatively and quantitatively analyzed by fluorescence microscopy (TE2000; Nikon; Kyoto, Japan) and flow cytometry (FACS; BD Biosciences; San Jose, CA, USA), respectively. The transfection medium was replaced with normal medium and then replaced with Tf-PL-GFP and GL-GFP for uptake studies. The cells were washed three times with PBS and fixed in 4 % paraformaldehyde, then DAPI stained the nuclei and finally subjected to fluorescence microscopic observation.

#### ***In vitro*cytotoxicity assays**

SMMC-7721 cells were seeded in triplicate in a 96-well plate (5 × 10^3^ cells/well). After 24 hours, the medium was replaced with 100 µL of complete growth medium containing different concentrations of transferrin liposomes and incubated for an additional 24, 48, 72 hours. Cells that were not exposed to the transfected protein liposomes were used as controls. Cell viability was measured by the MTT assay according to the manufacturer’s instructions.

#### Cell migration assay

SMMC-7721 cell migration was measured using a transwell assay kit (Corning Life Sciences; Tewksbury, MA, USA) with 8 µm pores as previously described. SMMC-7721 cells were suspended in a serum-free medium containing the gene preparation and DMEM supplemented with 10 % FBS as a chemoattractant. Cells migrated after 16 hours were stained with 0.1 % crystal violet and counted from 5 randomly selected regions under an inverted microscope.

#### Wound healing assay

About 5 × 10^5^ SMMC-7721 cells were added into a 6-well cell culture plate. The next day, scrape the cells straight with 1 mL tip, and wash off the suspended cells with PBS, and then serum-free medium was added. Incubate 6-well cell culture plate at 37 ℃ within 5 % CO_2_ cell incubator. Use an inverted microscope to observe cell repair and take pictures after 24 h.

#### Cell cycle and apoptosis analysis

Human liver cancer SMMC-7721 cells were inoculated in a 6-well plate with 1.5 × 10^5^ cells /2 mL per well and cultured overnight at 37 °C in 5 % CO_2_ incubator. The cells were then cultured in 2.5 µg/mL AChE, GL/AChE, and Tf-PL/AChE medium for 24, 48, and 72 h, and the control group was set with medium only.

1.  Cell cycle detection: Cells were collected, washed twice with pre-cooled PBS at 4 °C, added with pre-cooled 70 % ethanol, and fixed at 4 °C for 24 h. The fixed cells were washed twice with pre-cooled PBS at 4 °C, then 0.2 mL PI staining solution (200 µg/mL) was added. The fixed cells were bathed at 37 °C for 20 min and then detected by flow cytometry.

2.  Apoptosis detection: The cells were collected and washed with 4 °C pre-cooled PBS twice. The cells were resuspended with 200 µL binding buffer. Annexin V-FITC (5 µL) and PI (10 µL) were added.

Flow cytometry analysis was performed using a BD flow cytometer (Calibur; USA) to assess cell cycle distribution and apoptosis.

#### ***In vivo*imaging of mice inoculated with SMMC-7721**

SMMC-7721 cells (2 × 10^6^) were injected subcutaneously into the right dorsal skin of 6-week-old female BALB/c nude mice. The tumor is allowed to grow for 10 days to about 100 mm3 after inoculation. Mice were randomly divided into three groups, a blank control group, a non-targeted treatment group, and a targeted treatment group, n = 3 in each group. 200 µL of physiological saline containing Cy5.5, Cy5.5-labeled non-targeted liposome (GL), Cy5.5-labeled transferrin liposome (Tf-PL) were injected at a dose of 100 µg/kg Cy5, respectively. Images were taken at 24 and 72 h after injection using the MAESTRO *in vivo* imaging system (Cambridge Research & Instrumentation; Hopkinton, MA, USA). The mice were then harvested and the heart, liver, spleen, lung, kidney, and tumor were dissected, washed with saline, and imaged using the MAESTRO *in vitro* imaging system.

#### **Evaluation of antitumor efficiency and safety*in vivo***

Mice bearing SMMC-7721 liver cancer were established as described above and randomly divided into 4 groups (n = 6 per group), control, AChE, GL/AChE and Tf-PL/AChE. Mice were injected intravenously with saline (control) or therapeutic agent 50 µg/kg at 10, 12, 14, 16, 18, 20, 22, 24, and 26 days after implantation. The anti-tumor efficiency was determined according to the tumor volume using the following formula: larger diameter × (smaller diameter/2)^2^. Systemic toxicities were assessed by monitoring body weight changes and nephrotoxicity.

#### HE Staining

For HE staining, tissues were fixed in 4 % paraformaldehyde for more than 24 h. Paraffin-embedded tissue sections (4 µm) were dewaxed and rehydrated. Hydration sections were stained with Mayer’s hematoxylin and eosin.

### Statistical analysis

For multiple comparisons, a one-way ANOVA test was performed. The t-test (two-tailed) was used for comparison between the two groups. Data are expressed as mean ± standard deviation (S.D.). Survivors were estimated using a log-rank test. *p < 0.05, **p < 0.01, ***p < 0.001.

## Supplementary Information

**Additional file 1: Figure S1.** Correlation between the expression level of transferrin receptor and liver cancer progression.** Figure S2.** The survival rate of patients with high and low TfR expression level.** Figure S3.** The expression level of ACh in liver cancer.** Figure S4. **The expression level of AchE in liver cancer.** Figure S5.** The expression level of TfR in different cell lines.** Figure S6.** 1H-NMR spectrum of Tf-GHDC.** Figure S7.** Standard curve of Transferrin (Tf).** Figure S8**. The stability analysis of the different AChE formulations.** Figure S9.** Cytotoxicity of the prepared proteoliposomes.** Figure S10.** Subcellular localization analysis of Tf-PL.** Figure S11.** Effect on SMMC-7721 cell proliferation of ACh and AChE.** Figure S12.** In vitro cell migration of AChE treatment study.** Figure S13.** In vitro wound healing experiment of AChE treatment study.** Figure S14.** Photographic images of dissected tumor tissues.** Table S1.** Influence of different proportion of GHDC: Chol on liposome particle size.** Table S2.** Effects of different proportions of Tf-GHDC: Chol on the particle size of liposomes.** Table S3.** Effects of different proportion of GHDC: Chol: AChE on liposomal-loaded genes.** Table S4.** Effects of different proportions of Tf-GHDC: Chol: Ache on liposomal - loaded genes.** Table S5. **Optimization of Tf-GHDC: Chol: AChE ratio on lipoplastological influence.

## Data Availability

All data generated or analyzed during this study are included in this published article.

## References

[1] Moris D, Rahnemaiazar AA, Zhang X, Ntanasisstathopoulos I, Tsilimigras DI, Chakedis J, Argyrou C, Fung JJ, Pawlik TM (2017). Program death-1 immune checkpoint and tumor microenvironment in malignant liver tumors. Surgic Oncol Oxford.

[2] Nio K, Yamashita T, Kaneko S (2017). The evolving concept of liver cancer stem cells. Mol Cancer.

[3] Raouf S, Weston C, Yucel N, Iorns E, Gunn W, Tan F, Lomax J, Errington T (2015). Senescence surveillance of pre-malignant hepatocytes limits liver cancer development. Nature.

[4] Alexandrov LB, Ju YS, Haase K, Van Loo P, Martincorena I, Nik-Zainal S, Totoki Y, Fujimoto A, Nakagawa H, Shibata T (2016). Mutational signatures associated with tobacco smoking in human cancer. Science.

[5] Connor F, Rayner TF, Aitken SJ, Feig C, Lukk M, Santoyo-Lopez J, Odom DT (2018). Mutational landscape of a chemically-induced mouse model of liver cancer. J Hepatol.

[6] De Marzo AM, Platz EA, Sutcliffe S, Xu J, Grönberg H, Drake CG, Nakai Y, Isaacs WB, Nelson WG (2007). Inflammation in prostate carcinogenesis. Nat Rev Cancer.

[7] Mak KY, Rajapaksha IG, Angus PW, Herath CB (2017). The adeno-associated virus-a safe and effective vehicle for liver-specific gene therapy of inherited and non-inherited diseases. Curr Gene Therapy.

[8] Samson A, Bentham MJ, Scott K, Nuovo G, Bloy A, Appleton E, Adair RA, Dave R, Peckhamcooper A, Toogood G (2018). Oncolytic reovirus as a combined antiviral and anti-tumour agent for the treatment of liver cancer. Gut.

[9] Xia H, Hui KM (2017). Emergence of aspirin as a promising chemopreventive and chemotherapeutic agent for liver cancer. Cell Death Dis.

[10] Yang D, Luo W, Wang J, Zheng M, Liao XH, Zhang N, Lu W, Wang L, Chen AZ, Wu WG (2017). A novel controlled release formulation of the Pin1 inhibitor ATRA to improve liver cancer therapy by simultaneously blocking multiple cancer pathways. J Control Release.

[11] Klinghoffer RA, Bahrami SB, Hatton BA, Frazier JP, Morenogonzalez A, Strand AD, Kerwin WS, Casalini JR, Thirstrup DJ, You S (2015). A technology platform to assess multiple cancer agents simultaneously within a patient’s tumor. Sci Transl Med.

[12] Mcdermott U, Ames RY, Iafrate AJ, Maheswaran S, Stubbs H, Greninger P, Mccutcheon K, Milano R, Tam A, Lee DY (2009). Ligand-dependent PDGF receptor-alpha activation sensitizes rare lung cancer and sarcoma cells to PDGF receptor kinase inhibitors. Can Res.

[13] Tardieu M, Zérah M, Gougeon ML, Ausseil J, De BS, Husson B, Zafeiriou D, Parenti G, Bourget P, Poirier B (2017). Intracerebral gene therapy in children with mucopolysaccharidosis type IIIB syndrome: an uncontrolled phase 1/2 clinical trial. Lancet Neurol.

[14] Chen L, Simpson JD, Fuchs AV, Rolfe BE, Thurecht KJ (2017). Effects of surface charge of hyperbranched polymers on cytotoxicity, dynamic cellular uptake and localization, hemotoxicity, and pharmacokinetics in mice. Mol Pharm.

[15] Tang XH, Xie P, Ding Y, Chu LY, Hou JP, Yang JL, Song X, Xie YM (2010). Synthesis, characterization, and in vitro and in vivo evaluation of a novel pectin-adriamycin conjugate. Bioorg Med Chem.

[16] Dowdy SF (2017). Overcoming cellular barriers for RNA therapeutics. Nat Biotechnol.

[17] Herrmann A, Nagao T, Zhang C, Lahtz C, Li YJ, Yue C, Mülfarth R, Yu H (2019). An effective cell-penetrating antibody delivery platform. JCI Insight.

[18] Wang X, Xia Y (2019). Anti-double stranded DNA antibodies: origin, pathogenicity, and targeted therapies. Front Immunol.

[19] Gao SX, Cai SF, Rui-An XU (2017). The strategies of targeting therapy of hepatocellular carcinoma by adeno-associated virus. Acta Pharm Sinica.

[20] Tesic N, Kamensek U, Sersa G, Kranjc S, Stimac M, Lampreht U, Preat V, Vandermeulen G, Butinar M, Turk B (2015). Endoglin (CD105) silencing mediated by shRNA under the control of endothelin-1 promoter for targeted gene therapy of melanoma. Molecular Therapy Nucleic Acids.

[21] Whitehead KA, Dorkin JR, Vegas AJ, Chang PH, Veiseh O, Matthews J, Fenton OS, Zhang Y, Olejnik KT, Yesilyurt V (2014). Degradable lipid nanoparticles with predictable in vivo siRNA delivery activity. Nat Commun.

[22] Li G, Yin X, Wei W, Hong M (2017). Preparation of graphene via liquid-phase exfoliation with high gravity technology from edge-oxidized graphite. Coll Surf a Physicochem Eng Aspects.

[23] Von WLK, Weske S, Keul P, Peters S, Baba HA, Heusch G, GrãLer MH, Levkau B (2017). Hepatocyte nuclear factor 1A deficiency causes hemolytic anemia in mice by altering erythrocyte sphingolipid homeostasis. Blood.

[24] Gudjonsson A, Lysén A, Balan S, Sundvoldgjerstad V, Arnoldschrauf C, Richter L, Bækkevold ES, Dalod M, Bogen B, Fossum E (2017). Targeting influenza virus hemagglutinin to Xcr1(+) dendritic cells in the absence of receptor-mediated endocytosis enhances protective antibody responses. J Immunol.

[25] Singh Y (2008). Recent trends in targeted anticancer prodrug and conjugate design. Curr Med Chem.

[26] Wang J, Li W, Zhang L, Ban L, Chen P, Du W, Feng X, Liu BF (2017). Chemically edited exosomes with dual ligand purified by microfluidic device for active targeted drug delivery to tumor cells. Acs Appl Mater Interfaces.

[27] Wang S, Li C, Ying M, Min Q, Jiang H, Du Y, Huang R, Yi W (2017). MemHsp70 receptor-mediated multifunctional ordered mesoporous carbon nanospheres for photoacoustic imaging-guided synergistic targeting trimodal therapy. Acs Biomater Sci Eng.

[28] Yin T, Liu J, Zhao Z, Zhao Y, Dong L, Meng Y, Zhou J, Huo M (2017). Redox sensitive hyaluronic acid-decorated graphene oxide for photothermally controlled tumor‐cytoplasm‐selective rapid drug delivery. Adv Func Mater.

[29] Akinc A, Maier MA, Manoharan M, Fitzgerald K, Jayaraman M, Barros S, Ansell S, Du X, Hope MJ, Madden TD (2019). The Onpattro story and the clinical translation of nanomedicines containing nucleic acid-based drugs. Nat Nanotechnol.

[30] Hoy SM (2018). Patisiran: first global approval. Drugs.

[31] Bennett CF (2019). Therapeutic antisense oligonucleotides are coming of age. Annu Rev Med.

[32] Giannetti AM, Halbrooks PJ, Mason AB, Vogt TM, Enns CA, Björkman PJ (2005). The molecular mechanism for receptor-stimulated iron release from the plasma iron transport protein transferrin. Structure.

[33] Kleven MD, Jue S, Enns CA (2018). The transferrin receptors, TfR1 and TfR2, bind transferrin through differing mechanisms. Biochemistry.

[34] Yoshinaga M, Nakatsuka Y, Vandenbon A, Ori D, Uehata T, Tsujimura T, Suzuki Y, Mino T, Takeuchi O (2017). Regnase-1 maintains iron homeostasis via the degradation of transferrin receptor 1 and Prolyl-Hydroxylase-Domain-Containing Protein 3 mRNAs. Cell Reports.

[35] Bowers AJ, Scully S, Boylan JF (2003). SKIP3, a novel Drosophila tribbles ortholog, is overexpressed in human tumors and is regulated by hypoxia. Oncogene.

[36] Goswami U, Dutta A, Raza A, Kandimalla R, Kalita S, Ghosh SS, Chattopadhyay A (2017). Transferrin–Copper nanocluster–doxorubicin nanoparticles as targeted theranostic cancer nanodrug. Acs Appl Mater Interfaces.

[37] Hall PA, Todd CB, Hyland PL, Mcdade SS, Grabsch H, Dattani M, Hillan KJ, Russell SEH (2005). The septin-binding protein anillin is overexpressed in diverse human tumors. Clin Cancer Res.

[38] Sokolowska-Wedzina A, Chodaczek G, Chudzian J, Borek A, Zakrzewska M, Otlewski J (2017). High-affinity internalizing human scFv-Fc antibody for targeting FGFR1-overexpressing Lung cancer. Mol Cancer Res Mcr.

[39] Macdonald E, Brown L, Selvais A, Liu H, Waring T, Newman D, Bithell J, Grimes D, Urbé S, Clague MJ (2018). HRS–WASH axis governs actin-mediated endosomal recycling and cell invasion. J Cell Biol.

[40] Sakurai K, Sohda T, Ueda S, Tanaka T, Hirano G, Yokoyama K, Morihara D, Aanan A, Takeyama Y, Irie M (2014). Immunohistochemical demonstration of transferrin receptor 1 and 2 in human hepatocellular carcinoma tissue. Hepato-gastroenterology.

[41] Avci ME, Keskus AG, Targen S, Isilak ME, Ozturk M, Atalay RC, Adams MM, Konu O (2018). Development of a novel zebrafish xenograft model in ache mutants using liver cancer cell lines. Sci Rep.

[42] Zhao Y, Wang X, Wang T, Hu X, Hui X, Yan M, Gao Q, Chen T, Li J, Yao M (2011). Acetylcholinesterase, a key prognostic predictor for hepatocellular carcinoma, suppresses cell growth and induces chemosensitization. Hepatology.

[43] Basiri A, Xiao M, Mccarthy A, Dutta D, Byrareddy SN, Conda-Sheridan M (2017). Design and synthesis of new piperidone grafted acetylcholinesterase inhibitors. Bioorg Med Chem Lett.

[44] Ismaili L, Refouvelet B, Benchekroun M, Brogi S, Brindisi M, Gemma S, Campiani G, Filipic S, Agbaba D, Esteban G (2017). Multitarget compounds bearing tacrine- and donepezil-like structural and functional motifs for the potential treatment of Alzheimer’s disease. Prog Neurobiol.

[45] Pascale RM, De Miglio MR, Muroni MR, Simile MM, Daino L, Seddaiu MA, Pusceddu S, Gaspa L, Calvisi D, Manenti G, Feo F (1998). Transferrin and transferrin receptor gene expression and iron uptake in hepatocellular carcinoma in the rat. Hepatology.

[46] Hong Y, Yang J, Shen X, Zhu H, Sun X, Wen X, Bian J, Hu H, Yuan L, Tao J (2013). Sinomenine hydrochloride enhancement of the inhibitory effects of anti-transferrin receptor antibody-dependent on the COX-2 pathway in human hepatoma cells. Cancer Immunol Immunother.

