# Supplementary material for: Protein liposomes-mediated targeted acetylcholinesterase gene delivery for effective liver cancer therapy
Source: J Nanobiotechnology. 2021 Jan 22;19:31. doi: 10.1186/s12951-021-00777-9 (PMC7821407; doi:10.1186/s12951-021-00777-9)
Supplement: Supplementary file 1 — Additional file 1: Figure S1. Correlation between the expression level of transferrin receptor and liver cancer progression. Figure S2. The survival rate of patients with high and low TfR expression level. Figure S3. The expression level of ACh in liver cancer. Figure S4. The expression level of AchE in liver cancer. Figure S5. The expression level of TfR in different cell lines. Figure S6. 1H-NMR spectrum of Tf-GHDC. Figure S7. Standard curve of Transferrin (Tf). Figure S8. The stability analysis of the different AChE formulations. Figure S9. Cytotoxicity of the prepared proteoliposomes. Figure S10. Subcellular localization analysis of Tf-PL. Figure S11. Effect on SMMC-7721 cell proliferation of ACh and AChE. Figure S12. In vitro cell migration of AChE treatment study. Figure S13. In vitro wound healing experiment of AChE treatment study. Figure S14. Photographic images of dissected tumor tissues. Table S1. Influence of different proportion of GHDC: Chol on liposome particle size. Table S2. Effects of different proportions of Tf-GHDC: Chol on the particle size of liposomes. Table S3. Effects of different proportion of GHDC: Chol: AChE on liposomal-loaded genes. Table S4. Effects of different proportions of Tf-GHDC: Chol: Ache on liposomal - loaded genes. Table S5. Optimization of Tf-GHDC: Chol: AChE ratio on lipoplastological influence. [file 12951_2021_777_MOESM1_ESM.docx]

**Additional file**

**Additional file 1: Figures**


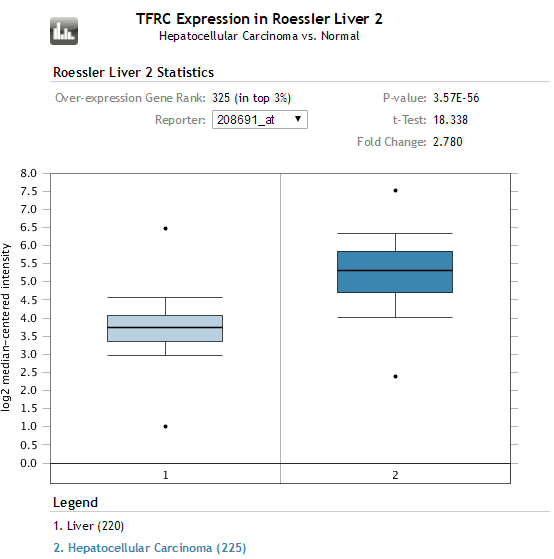


**Figure S1.** **Correlation between the expression level of transferrin receptor and liver cancer progression.**

The expression level of transferrin receptor in liver cancer tissue is higher than lover tissue.
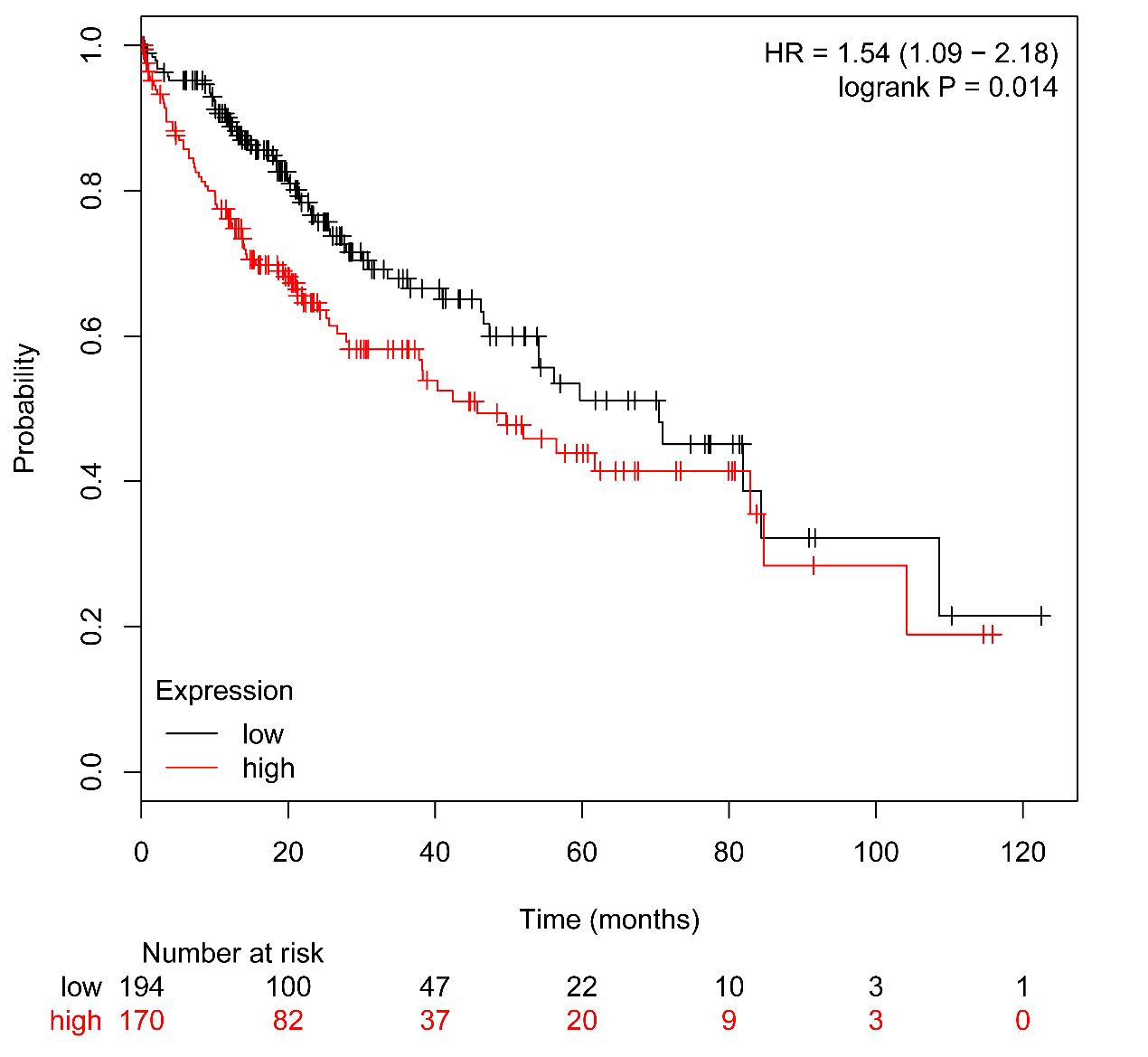


**Figure S2.** **The survival rate of patients with high and low TfR expression level.**

Patients with high levels of TfR expression had relatively shorter survival period.
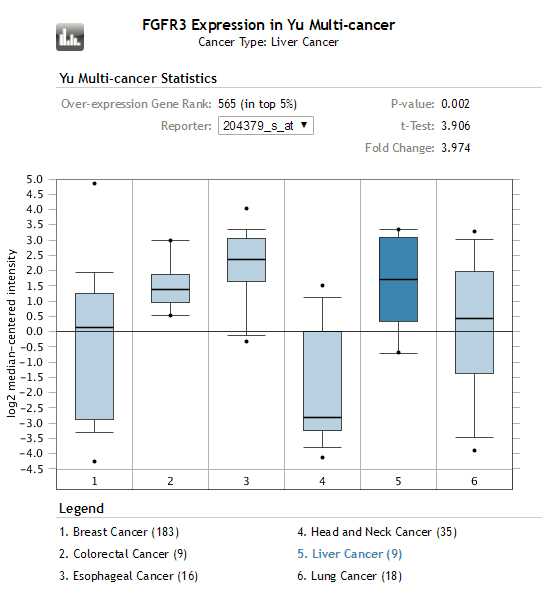


**Figure S3.** **The expression level of ACh in liver cancer.**

The expression level of ACh in the liver cancer tissue was relatively higher than that of normal tissue.


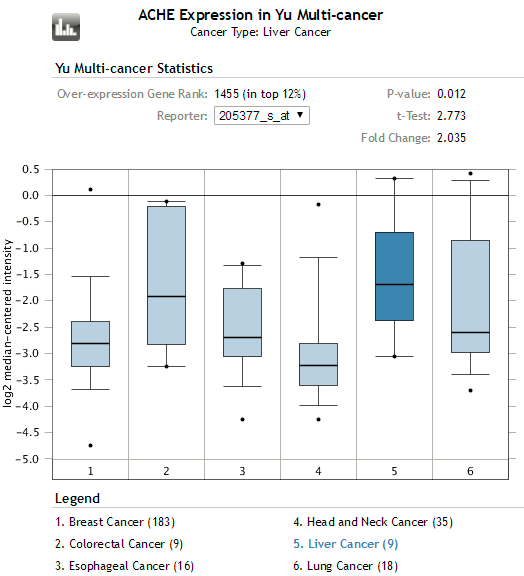


**Figure S4.** **The expression level of AchE in liver cancer.**

The expression level of AChE in the liver cancer tissue is relatively lower than that of normal tissue.


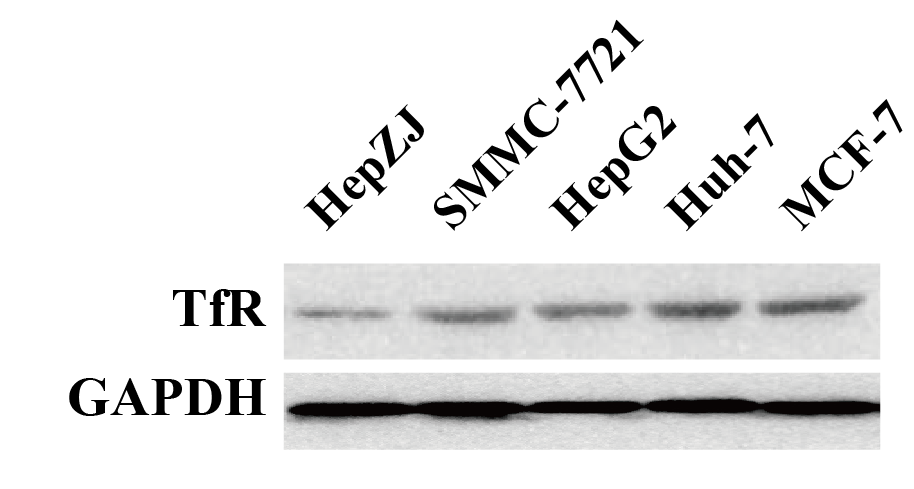


**Figure S5.** **The expression level of TfR in different cell lines.**


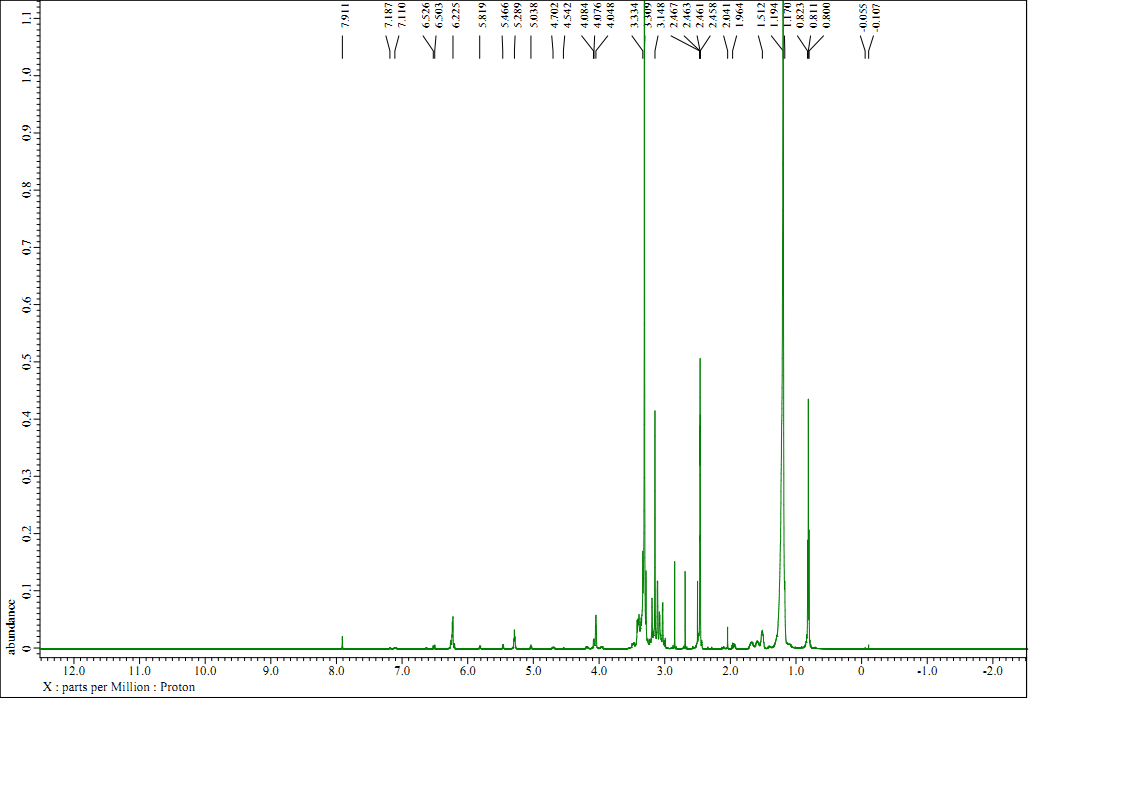


**Figure S6.** **^1^H-NMR spectrum of Tf-GHDC.**

Tf-GHDC were dissolved in D_2_O for ^1^H-NMR spectrum.


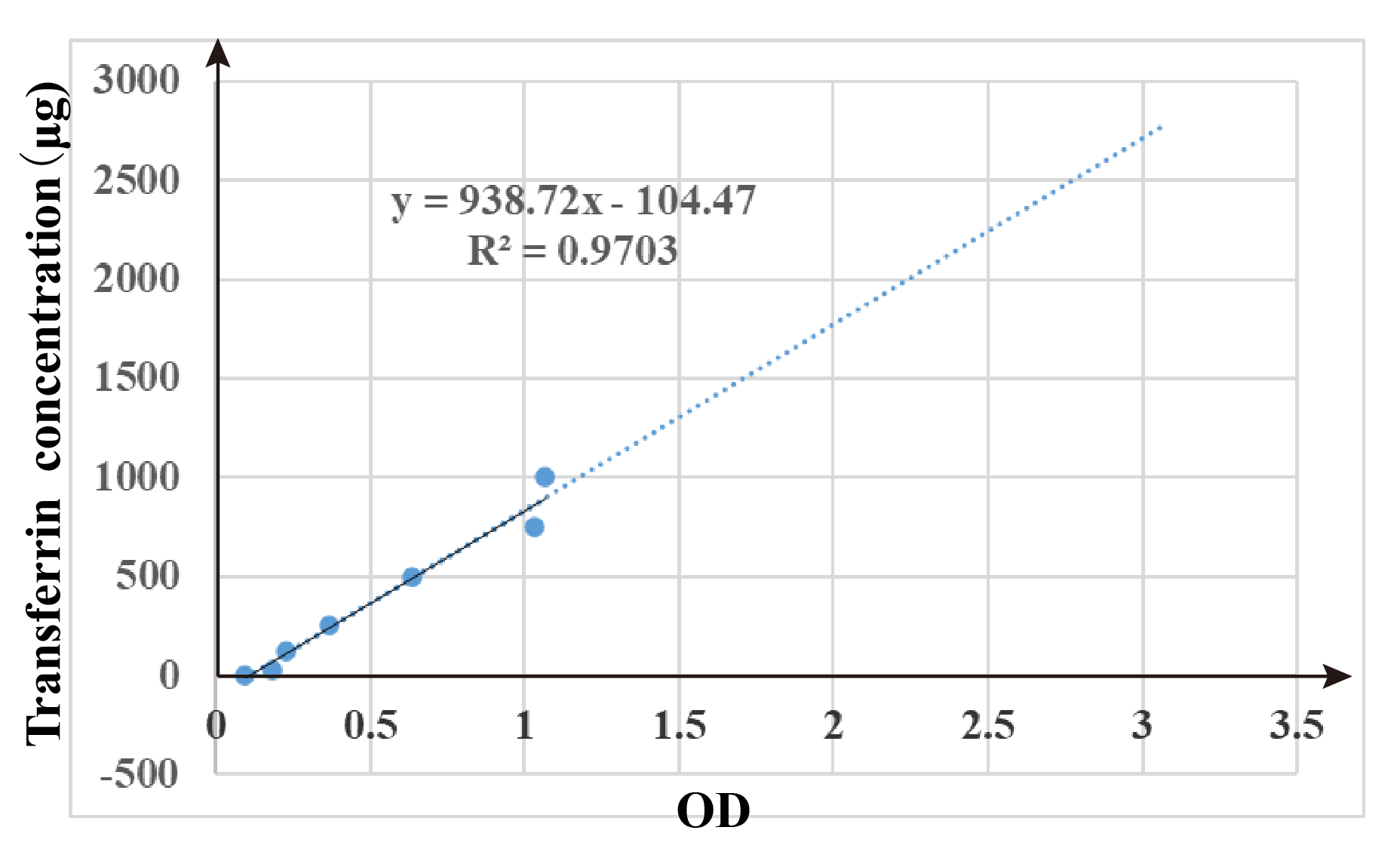


**Figure S7. Standard curve of Transferrin (Tf).**


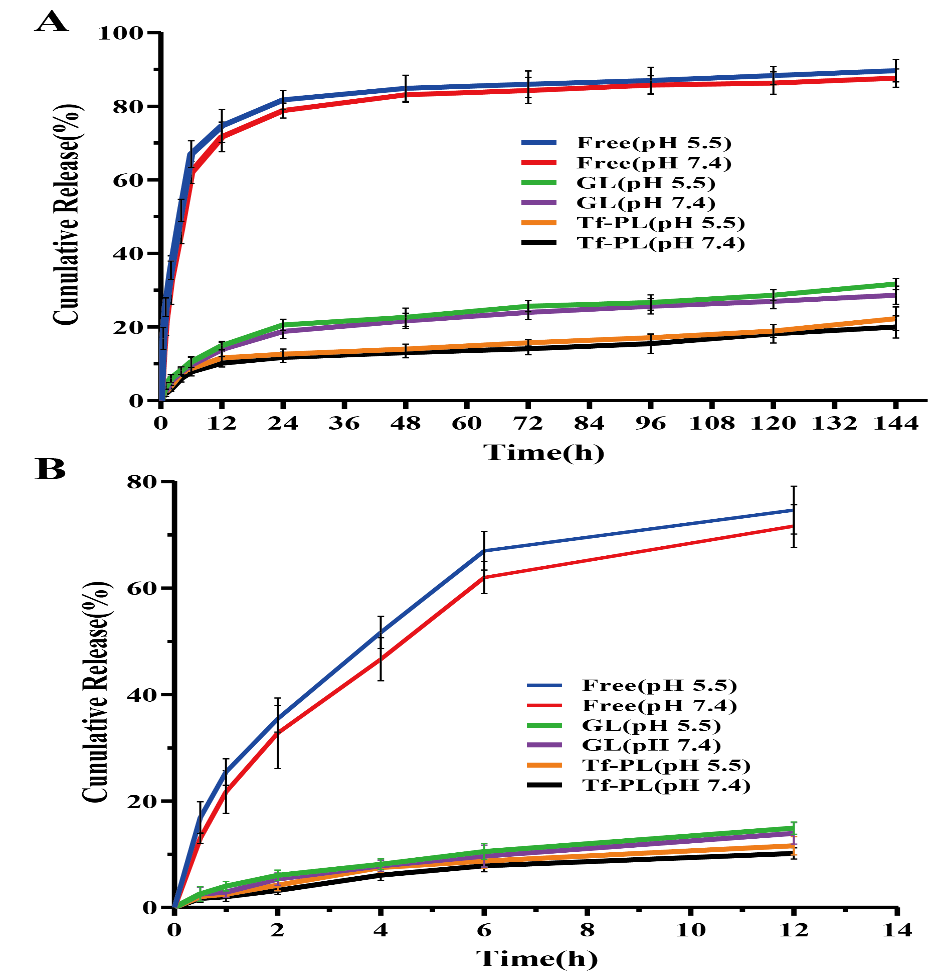


**Figure S8. The stability analysis of the different AChE formulations**.

A: Release of the whole observation points；B: Release of the observation points within 12 h of AChE gene.


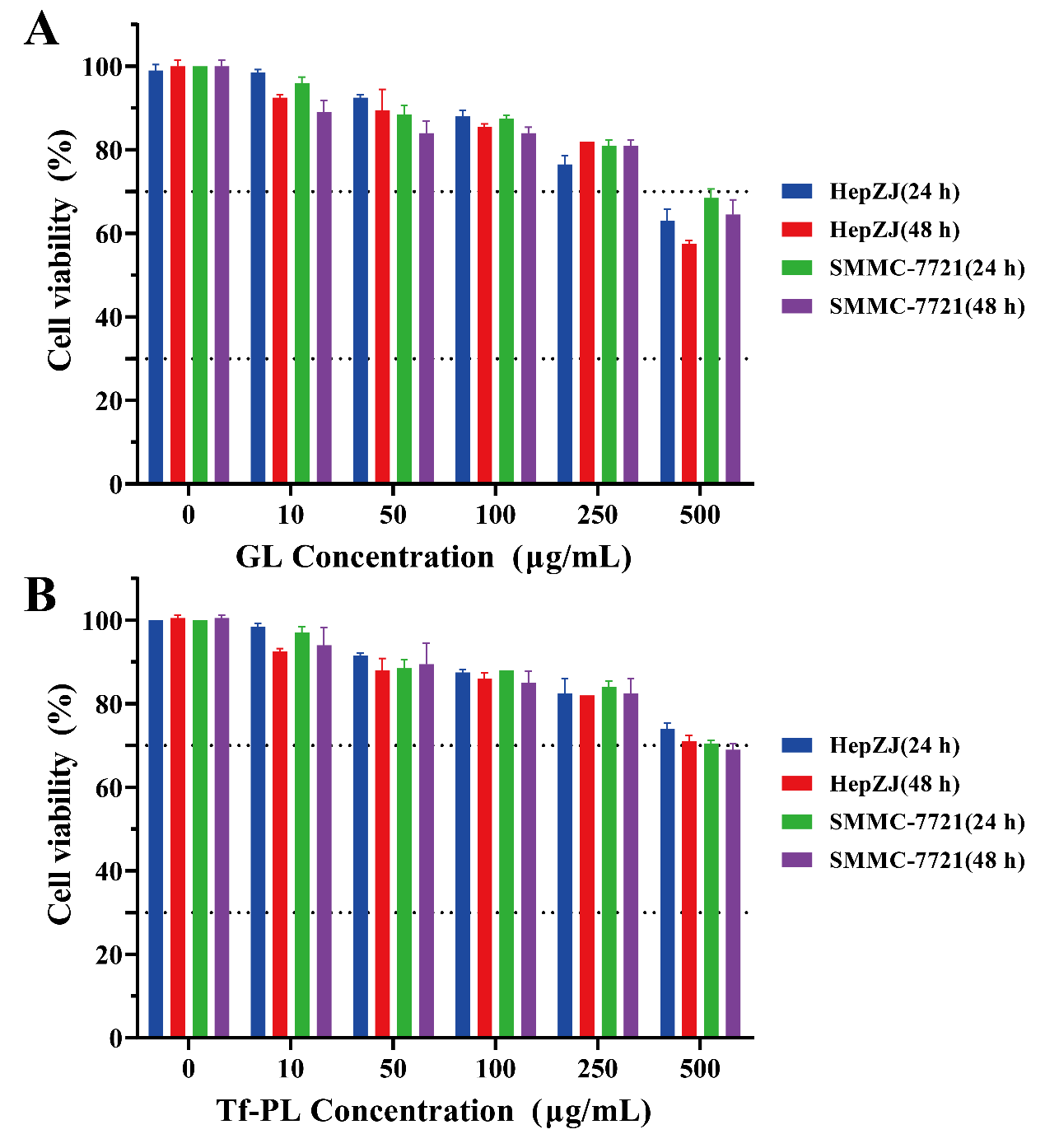


**Figure S9. Cytotoxicity of the prepared proteoliposomes.**

A: GL, B: Tf-PL.


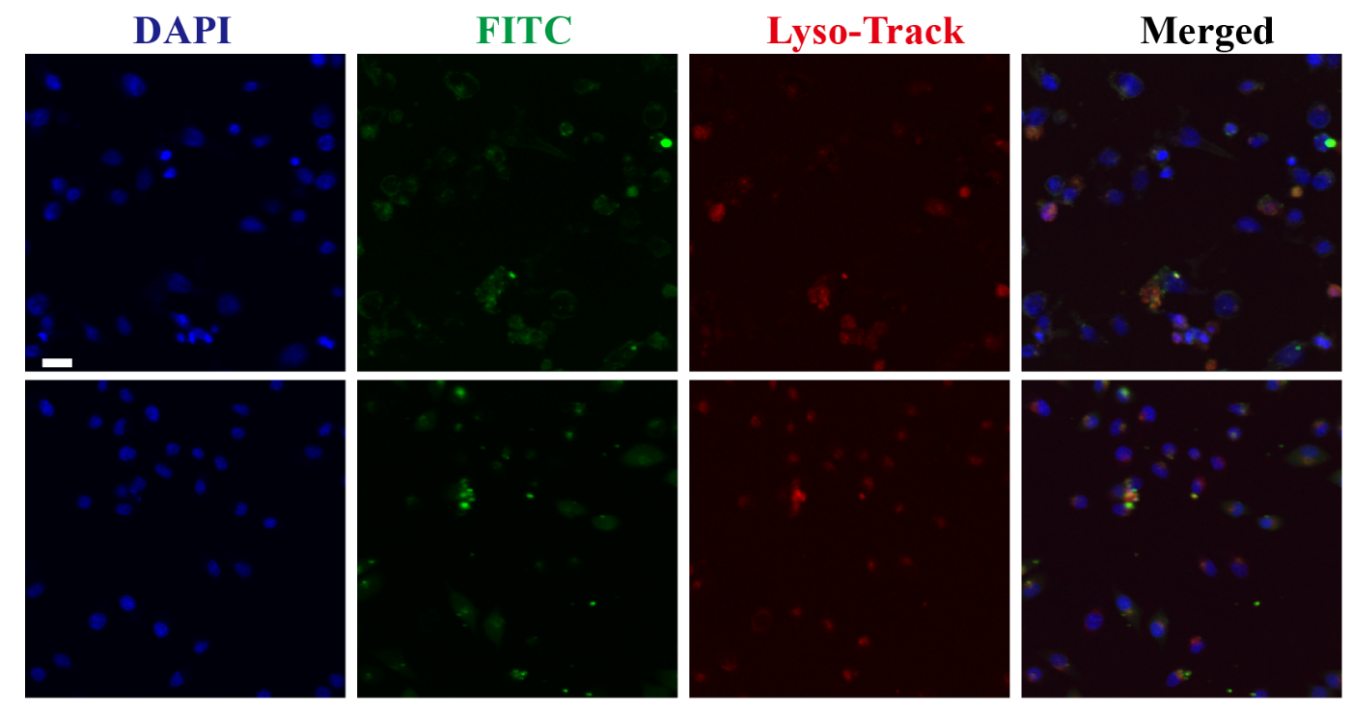


**Figure S10. Subcellular localization analysis of Tf-PL.**

Scale bar =10 μm.


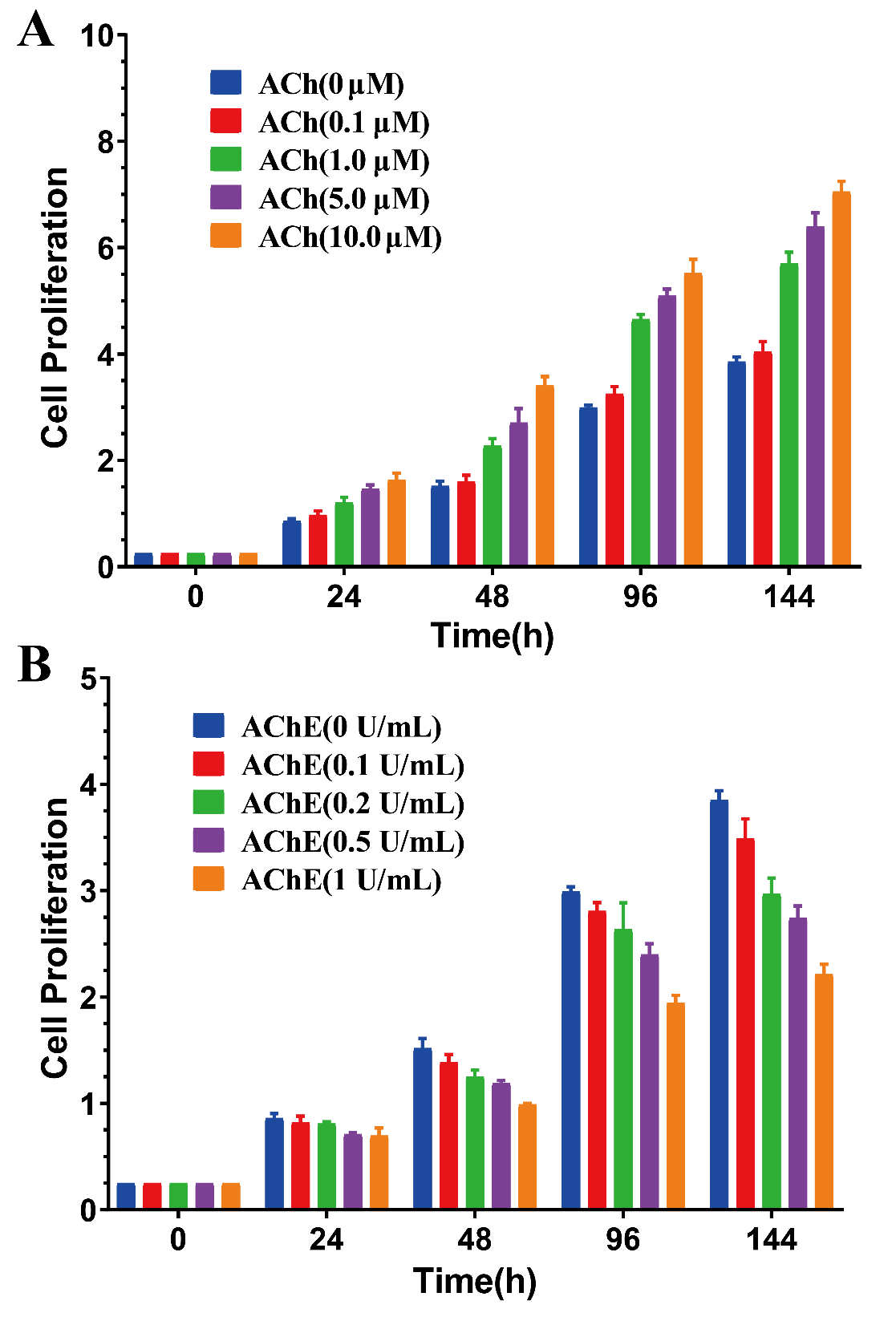


**Figure S11.** **Effect on SMMC-7721 cell proliferation of ACh and AChE.**

A: SMMC-7721 cell proliferation under different ACh concentration;

B: SMMC-7721 cell proliferation under different AChE concentration.

**
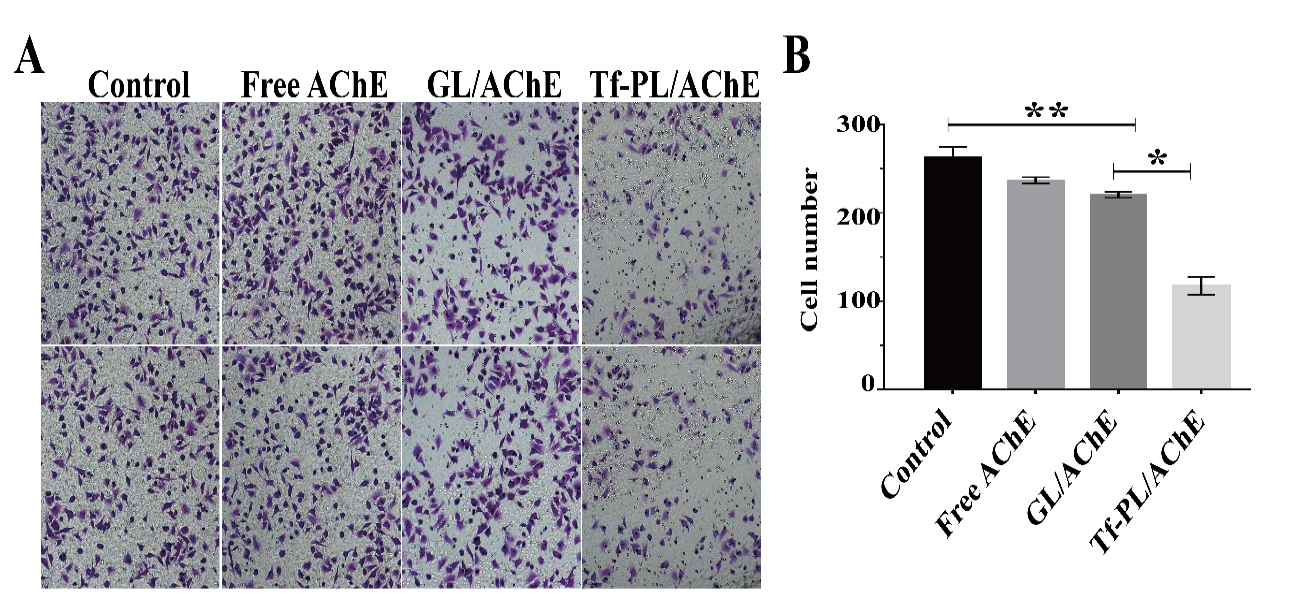
**

**Figure S12. In vitro cell migration of AChE treatment study.**

A: Inhibition of cell migration in SMMC-7721 cells following AChE treatment representative images of the Transwell assay showing the inhibitory effect of AChE on cell migration following treatment with Control, Free AChE, GL/AChE, Tf-PL/AChE for 2 h at an equivalent AChE concentration of 2.5 μg/mL. B: Quantiﬁation showing the migration inhibition rates in SMMC-7721 cells following the treatments.


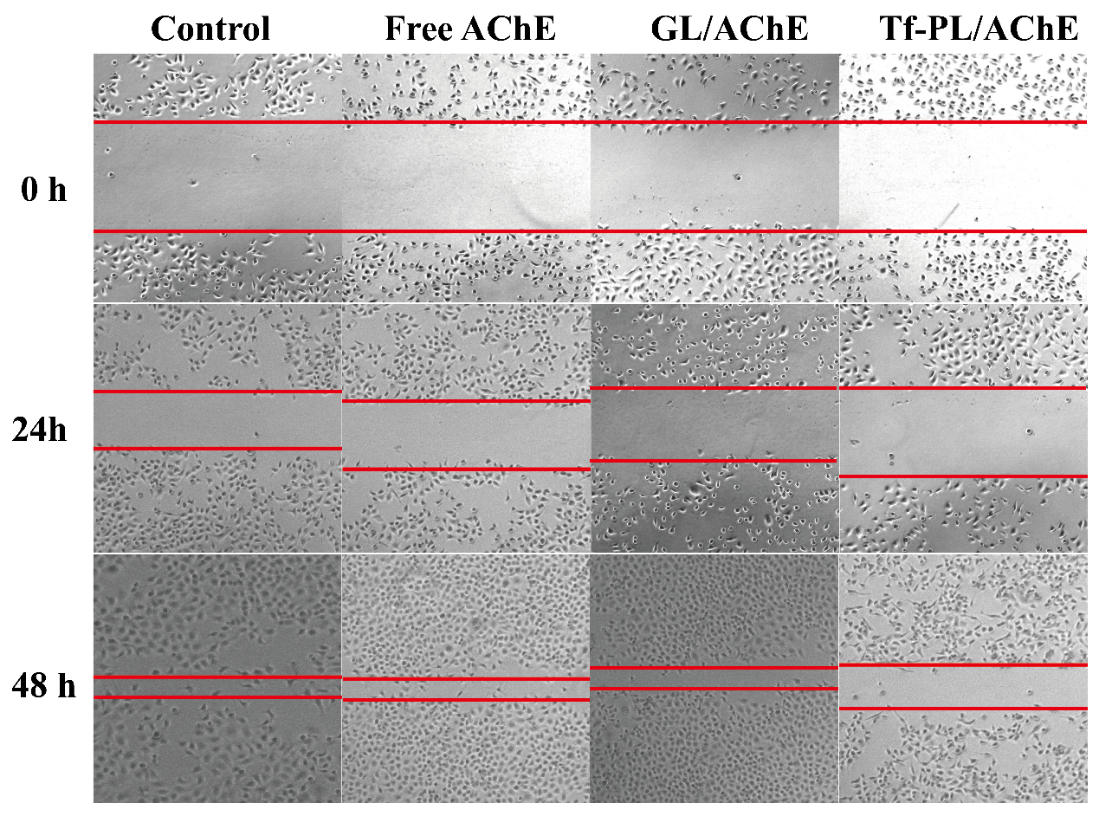


**Figure S13.** **In vitro wound healing experiment of AChE treatment study.**


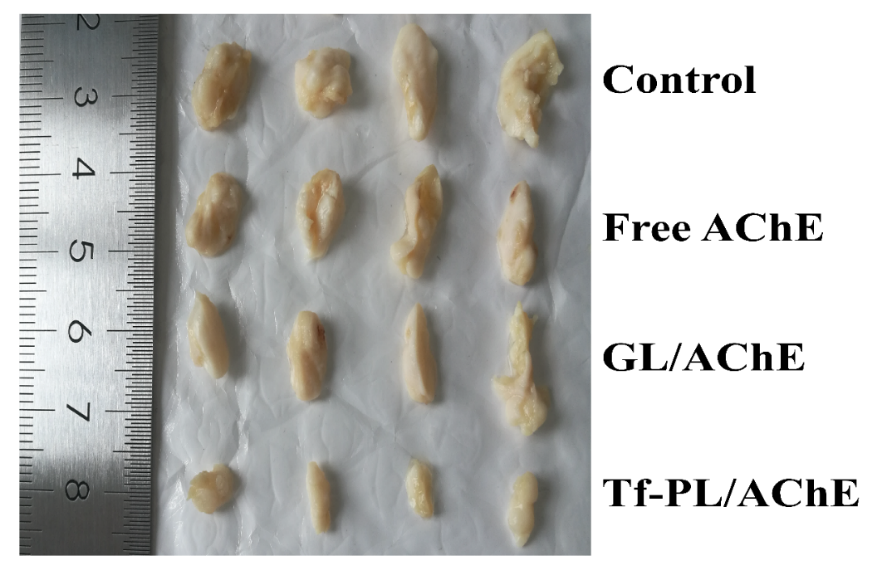


**Figure S14.** **Photographic images of dissected tumor tissues**.

**Additional file 1: Tables**

**Analysis of physical and chemical properties of liposomes**

**Table S1. Influence of different proportion of GHDC: Chol on liposome particle size**

| GHDC/Chol (W/W) | Mean diameter (nm) |
| --- | --- |
| 1:0.1 | 69.1 |
| 1:0.2 | 80.3 |
| 1:0.4 | 113.0 |
| 1:0.6 | 154.0 |
| 1:0.8 | 208.5 |
| 1:1.0 | 344.6 |

**Table S2. Effects of different proportions of Tf-GHDC: Chol on the particle size of liposomes**

| Tf-GHDC/Chol (W/W) | Mean diameter (nm) |
| --- | --- |
| 1:0.1 | 71.0 |
| 1:0.2 | 83.0 |
| 1:0.4 | 121.0 |
| 1:0.6 | 174.4 |
| 1:0.8 | 231.3 |
| 1:1.0 | 374.0 |

**Table S3. Effects of different proportion of GHDC: Chol: AChE on liposomal-loaded genes**

| GHDC/Chol /Ache(W/W/W) | Mean diameter (nm) | DL% | EL% |
| --- | --- | --- | --- |
| 1:0.1:0.1 | 92.1 | 5.82 | 90.1 |
| 1:0.1:0.2 | 96.0 | 6.07 | 91.2 |
| 1:0.1:0.5 | 135.0 | 6.13 | 91.3 |
| 1:0.2:0.1 | 154.0 | 5.15 | 90.0 |
| 1:0.2:0.2 | 178.1 | 6.1 | 91.0 |
| 1:0.2:0.5 | 272.6 | 5.63 | 92.1 |

**Table S4. Effects of different proportions of Tf-GHDC: Chol: Ache on liposomal - loaded genes**

| Tf-GHDC/Chol /AChE(W/W/W) | Mean diameter (nm) | DL% | EL% |
| --- | --- | --- | --- |
| 1:0.1:0.1 | 101.0 | 5.3 | 89.7 |
| 1:0.1:0.2 | 113.0 | 6.31 | 94.3 |
| 1:0.1:0.5 | 121.0 | 6.49 | 94.1 |
| 1:0.2:0.1 | 170.4 | 5.45 | 91.3 |
| 1:0.2:0.2 | 191.3 | 5.4 | 94.3 |
| 1:0.2:0.5 | 304.0 | 5.43 | 93.7 |

**Table S5. Optimization of Tf-GHDC: Chol: AChE ratio on lipoplastological influence**

| Formulation | Size | PDI | Zeta potential | DL% | EE% | CE% |
| --- | --- | --- | --- | --- | --- | --- |
| GL | 99.82 ±2.2 | 0.118±0.02 | 27.3 ± 0.4 | 6.07±0.43 | 91.2±0.79 | - |
| Tf-PL | 112.9 ± 4.5 | 0.149±0.03 | 21.8 ± 0.5 | 6.31±0.32 | 94.3±1.01 | 88.7±2.31 |
